# Modeling Control of Invasive Fire Ants by Gene Drive

**DOI:** 10.1002/advs.202504653

**Published:** 2025-10-27

**Authors:** Yiran Liu, Samuel E. Champer, Benjamin C. Haller, Jackson Champer

**Affiliations:** ^1^ Center for Bioinformatics Center for Life Sciences School of Life Sciences Peking University Beijing 100871 China; ^2^ Department of Computational Biology Cornell University Ithaca NY 14853 USA

**Keywords:** colony structures, computational modeling, fire ants, gene drives, suppression

## Abstract

The fire ant *Solenopsis invicta* is characterized by aggressive behavior and exceptional invasive capabilities, rendering conventional control methods largely ineffective. Here, we consider homing suppression gene drive in fire ants by developing a spatially explicit model that incorporates both monogyne and polygyne colonies. Ants may present unique challenges for gene drive due to their colony structure and haplodiploidy. Results show that after an extended period of time, gene drive effectively eliminates polygyne colonies, but monogyne populations can persist at low level. Though standard suppression drives in haplodiploids have reduced power, new dominant‐sterile resistance or two‐target strategies, as well as drives that affect the colony structure, can restore high suppressive capability. Interspecific competition can also exert a positive effect on gene drive suppression, especially if released during an initial invasion, enabling native ants to successfully recolonize their original habitats. Further, we identified several gRNA targets in conserved female fertility genes that may support efficient, low‐resistance suppression drive designs. Overall, we conclude that while gene drive in fire ants may take place over extended time scales, its long‐term results, even with imperfect efficiency, are promising.

## Graphical abstract

The fire ant *Solenopsis invicta* is characterized by exceptional invasive capabilities, rendering conventional control ineffective. Here, we explore the use of homing suppression gene drive in fire ants by developing a spatially explicit model. Overall, these results show that target population suppression requires an extended time scale, but that gene drive can still be highly effective.

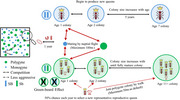

## 1 Introduction

The invasive fire ant, *Solenopsis invicta*, is a haplodiploid species notorious for its remarkable invasive capacity and resistance to control efforts. Accidentally introduced nearly a century ago from the Pantanal region in South America,^[^ ^1^ ^]^ fire ants have spread to numerous countries worldwide, including the United States,^[^ ^2^, ^3^, ^4^ ^]^ China,^[^ ^5^ ^]^ Australia,^[^ ^6^, ^7^ ^]^ and others.^[^ ^8^ ^]^ Recently, they were reported in Italy in 2023,^[^ ^9^ ^]^ marking the first recorded presence of this formidable species on the European continent. Damage caused by fire ants is extensive, encompassing ecological disruptions such as declines in native biodiversity,^[^ ^10^, ^11^ ^]^ agricultural loss through crop destruction,^[^ ^12^, ^13^ ^]^ and public health concerns due to their venomous stings and aggressive behavior.^[^ ^14^, ^15^, ^16^ ^]^ Various strategies have been explored for controlling fire ant populations. Bait traps have demonstrated some effectiveness in eliminating fire ant colonies, but their use poses risks to wildlife and domestic animals.^[^ ^17^ ^]^ Biological control involving pathogens and parasites has also been investigated.^[^ ^18^ ^]^ For example, an endoparasite nematode showed promising efficacy in the lab, but its performance was less effective in the field.^[^ ^18^ ^]^ Such challenges, including pesticide resistance, ecological side effects, and limited efficacy, highlight the ongoing difficulty in identifying a reliable and sustainable approach for managing fire ant populations.

Gene drive technology is a unique approach for managing agricultural pests or disease vectors. By biasing inheritance in its favor, a gene drive can increase in frequency within a population.^[^ ^19^, ^20^, ^21^ ^]^ By targeting fertility or viability genes, suppression gene drive systems can ultimately lead to the eventual elimination of the population. To date, suppression gene drives have been successfully implemented in the laboratory in various organisms, including fruit flies,^[^ ^22^ ^]^ spotted wing drosophila,^[^ ^23^ ^]^ mosquitoes,^[^ ^24^ ^]^ and medflies.^[^ ^25^ ^]^

Despite the growing interest in utilizing gene drive to manage invasive species,^[^ ^26^ ^]^ the application of this approach specifically to fire ant suppression has not been considered in detail. Fire ants are a haplodiploid species, meaning that males are haploid and possess only one set of chromosomes inherited from their mother, while females, which develop from fertilized eggs, are diploid. Haplodiploid species exhibit enhanced resilience to inbreeding depression due to their sex determination system,^[^ ^27^ ^]^ which both diminishes the suppressive effects of this phenomenon and increases the probability of resistance evolution in females.^[^ ^28^ ^]^ Previous studies have demonstrated that homing gene drive still has the potential to be an effective tool for controlling haplodiploid pests, though efficiency is lower than in diploids.^[^ ^29^, ^30^ ^]^ However, the unique lifecycle of fire ants presents additional challenges for the feasibility of gene drive applications. Specifically, the complex interactions between genetic inheritance and colony structure in fire ants may influence the success of gene drive strategies.

Colonies typically consist of a queen, female workers, and fertile ants (also named alates). Alates engage in nuptial flights, after which mated queens subsequently establish new colonies, though this process can be different for the polygyne social form (see below). Colonies are mostly stationary and can persist for several years. Females sterilized by a suppression drive could not produce workers and would thus not be competitive, potentially slowing suppression. Fire ant colonies can be classified into two distinct social forms. Those with a single reproductive queen are referred to as the monogyne form. Polygyne colonies have more than one queen. Fire ant social form proportions vary significantly across invaded regions. Surveys in Florida and southeastern U.S. states reveal stable, polygyne‐predominant populations,^[^ ^31^, ^32^ ^]^ while South America shows a monogyne‐dominant pattern.^[^ ^33^ ^]^ In China, social form proportions differ across distribution ranges.^[^ ^34^ ^]^ Monogyne and polygyne colonies substantially differ in behavioral ecology. For example, the queen in a monogyne colony, on average, has a longer life span than the smaller queens of polygyne colonies.^[^ ^35^ ^]^ Polygyne queens prefer to choose males in or near their own nest, and the colony reproduces by budding, while monogyne queens find males through nuptial flights, allowing for wider dispersal.^[^ ^36^ ^]^ Moreover, polygyne colonies exhibit reduced aggression toward other colonies with the polygyne social form.^[^ ^37^ ^]^ These social forms are closely associated with a “greenbeard” gene at a single genetic locus, which contains two alleles, *SB* and *Sb*, the latter of which yields a dominant polygyne phenotype.^[^ ^38^, ^39^ ^]^ Greenbeard in general refers to a detectable trait that results in holders of that trait acting to benefit each other, in this case, not attacking other colonies with the trait and removing female reproductive individuals lacking an *Sb* allele within polygyne colonies. Polygyne social forms manifest greater ant densities than monogyne colonies in many places (despite individually smaller workers), resulting in increased ecological perturbation and economic costs.^[^ ^40^ ^]^

Numerous modeling studies on suppression gene drive systems have revealed unexpected complexities when applied to spatially structured populations. These challenges often arise from the result of uneven suppression across the geographic distribution,^[^ ^41^ ^]^ which influences the spread of the drive and persistence of wild‐type.^[^ ^42^ ^]^ Environmental heterogeneity adds another layer of complexity, as variations in niche carrying capacity and local resource availability can influence the survival and reproductive success of individuals.^[^ ^43^, ^44^ ^]^ Together with other spatial dynamics,^[^ ^28^, ^45^ ^]^ these can significantly influence the propagation and effectiveness of gene drives and potentially lead to their failure. On the other hand, interactions with other species may also impact the outcome of gene drive strategies. In particular, the presence of competing or predator species could contribute to target species suppression.^[^ ^44^ ^]^

To determine the potential of gene drive for fire ant suppression, we model a homing suppression drive targeting a female fertility gene within a two‐dimensional spatial framework including fire ant lifecycle characteristics. To enhance suppression outcomes, we also consider several improved drive variants. These include dominant‐sterile resistance,^[^ ^46^ ^]^ a two‐target drive design,^[^ ^47^ ^]^ and hypothetical drive systems that affect fire ant social form. Recognizing that fire ants are an invasive species, our model also incorporates native ant populations to evaluate the potential role of native species in mitigating fire ant spread in conjunction with a gene drive. We found that while the complete suppression of fire ant populations required an extended time period, polygyne colonies were eliminated more rapidly. Greater suppression could be achieved with some of our improved drive strategies. Furthermore, the presence of native ant species also facilitated the suppression of fire ants, and releasing drive individuals during the invasion process could further accelerate target population elimination. Bioinformatic analysis shows that several promising female fertility target genes are highly conserved in fire ants and amenable to a gRNA multiplexing strategy. Overall, our findings provide a greater understanding of suppression gene drives in invasive fire ants and highlight the potential of utilizing native biodiversity in biocontrol strategies.

## 2 Experimental Section

### 2.1 Suppression Drive Strategy

Fire ants, as a haplodiploid species, have different chromosomal patterns than more common diploids. The egg‐laying queens are diploid, while the males usually only have one set of chromosomes, developing from unfertilized eggs. Accordingly, modeling was conducted for a homing suppression drive targeting a haplosufficient gene (where only one copy is required for normal organism function) that is essential for female fertility, which is the only type of powerful, self‐sustaining suppression drive that has shown to be viable for haplodiploids.^[^ ^30^ ^]^ The gene drive mechanism was implemented for each individual queen (the drive had no activity in haploid males). In germline cells of drive/wild‐type heterozygotes, the wild‐type allele was cleaved by CRISPR/Cas9, which was specifically guided by one or more guide RNAs (gRNAs). The cleaved chromosome then underwent homology‐directed repair, which resulted in the drive allele being copied to the wild‐type site (“drive conversion”). If a female inherits a drive allele from both parents, she will be homozygous and sterile. Because sterile females cannot produce workers, they will be unable to form a colony. With the growing fraction of sterile females as drive frequency increases, the population size will be decreased or even eliminated eventually if the fraction reaches a high enough level (based on the power of the drive).

If conversion does not occur, the wild‐type allele may instead mutate into a resistance allele, a process referred to as germline resistance allele formation (it also may remain uncut as a wild‐type allele and be passed on to progeny). Such resistance alleles cannot be recognized and cut by Cas9. Resistance may also arise when offspring inherit a wild‐type allele (from either parent) while the mother carries a drive allele. In such cases, any wild‐type allele in the offspring may be converted into a resistance allele due to the activity of the maternal deposited Cas9 and gRNA during early embryonic development (at a rate equal to the embryo resistance allele formation rate).

Resistance alleles are usually nonfunctional (referring to the disruption of the female fertility target gene). This kind of resistance still could cause female sterility but will slow down the drive process and reduce its power, especially if the drive has fitness costs. In another less frequent but more drastic outcome, the mutation will cause the suppression drive to fail almost immediately if the resistance allele preserves the function of the target gene. In such scenarios, females retain reproductive capacity due to the functional resistance allele, which will gain a selective advantage and rapidly outcompete the drive. However, experimental evidence demonstrates that targeting a highly conserved gene and gRNA multiplexing can mitigate the emergence of functional resistance^[^ ^24^, ^48^, ^49^, ^50^ ^]^ (and both together are likely sufficient to prevent it with good design in suppression drives). A synergistic combination of these offers a promising approach to prevent functional resistance formation. Hence, only nonfunctional resistance was considered in this model. However, a previous study found that haplodiploid species were somewhat more susceptible to the emergence of functional resistance alleles than diploids,^[^ ^30^ ^]^ reinforcing the need for careful experimental design for gene drives in haplodiploids.

Drive female heterozygotes can experience fitness costs due to unintended Cas9 activity (leakiness) in somatic cells. This off‐target activity disrupts wild‐type alleles in somatic cells, resulting in reduced fertility among female drive heterozygotes. This heterozygote fitness cost might also be caused by haploinsufficiency of target genes or even arise from desired germline activity, depending on the expression pattern of the female fertility target gene that was needed for fertility, or cleavage of the wild‐type allele in other ovary cells by Cas9.

### 2.2 Fire Ant Reproduction Model

**Figure 1:**
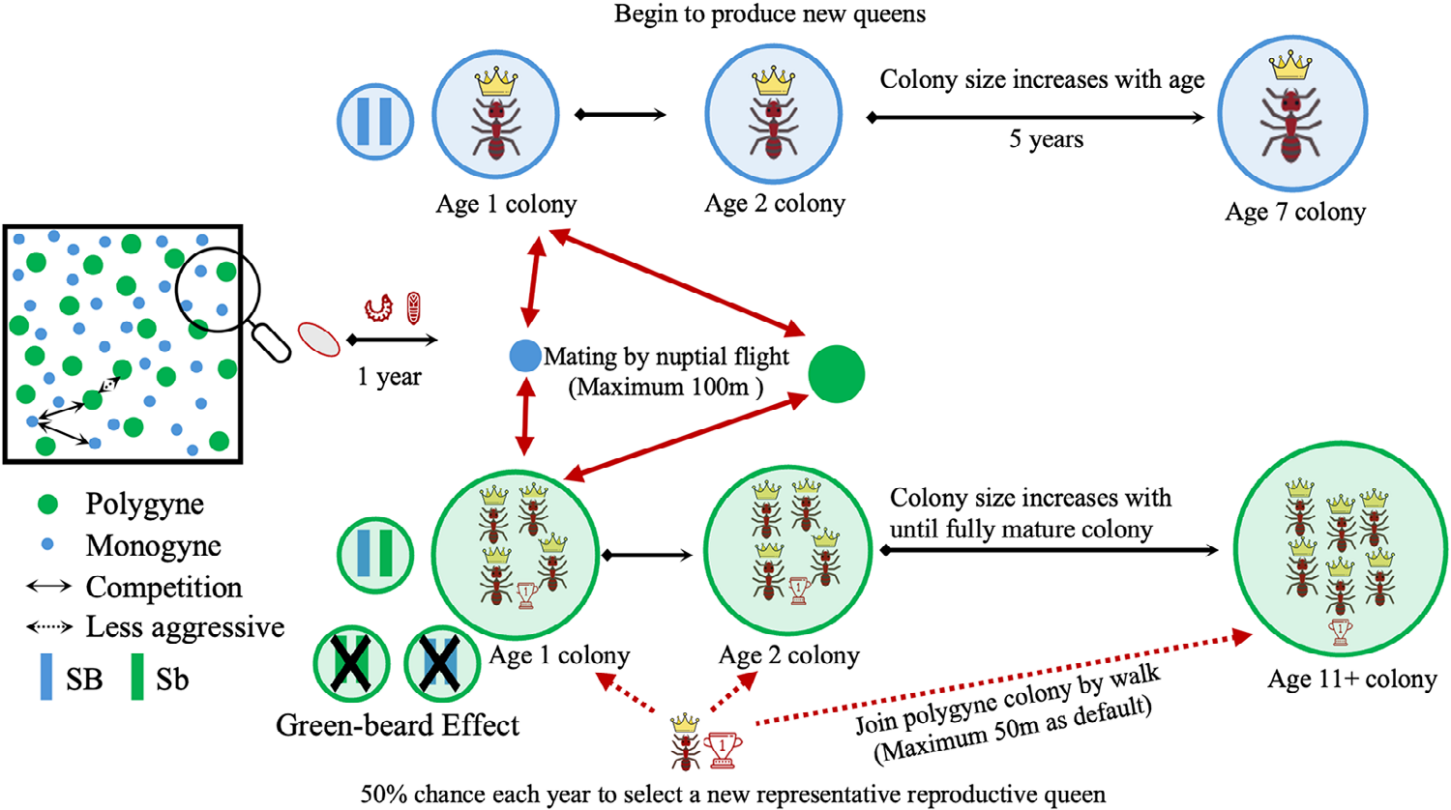
Diagram of the fire ant model. Two types of fire ant social forms are randomly distributed within the territory. Green dots represent polygyne colonies, while blue dots indicate monogyne colonies. Black arrows in the diagram illustrate competition between colonies, with dashed arrows representing weaker competition. The colonies are influenced by the greenbeard effect and Sb homozygote nonviability, meaning that polygyne colonies only give rise to SB/Sb heterozygous females. Red arrows indicate the nuptial flight and the distance for establishing a new colony, while red dashed arrows represent the shorter‐ranged expansion of polygyne colonies through budding.

The individual‐based, forward‐in‐time genetic simulation framework SLiM (version 4.3) was used to implement this model.^[^ ^51^ ^]^ The model was stochastic, with all simulations proceeding through random processes. In the fire ant model (**Figure** **1**), all colonies were represented by a single queen, which had a genotype for herself and a genotype for her previous mate. The model assumed a default population size of 50 000 monogyne colonies and 30 941 polygyne colonies (so that each social form occupied 50% of the territory at the start, see below), randomly distributed within a 1.85 km × 1.85 km territory (which usually could support 100 000 monogyne colonies at equilibrium in the model). Time steps were yearly, which represented a full reproductive cycle in fire ants.

Only colonies older than age 1 were capable of reproduction. In the program, age 1 queens could mate (selecting a mate from a colony capable of producing offspring) but could not produce any offspring until age 2. Typically, queens mate only once during their lifetime, which was reflected in this model.^[^ ^52^ ^]^ Note that fire ants mate before establishing a colony, but this took place after the first year of density‐dependent competition in this model to produce a nearly identical result (small differences in male availability might cause slightly different outcomes) with lower computational burden. This was possible because age‐0 colonies were too small to exert any significant competition on their neighbors. Age 1 new queens had up to 10 attempts to find a neighboring colony from which to take a mate, with the fitness of potential colonies determined by their genotype. Larger colonies also had a proportionately higher (based on their biomass) likelihood of being selected. Furthermore, polygyne colonies have a reduced rate of being selected, multipled by the “polygyne male rate” because polygyne colonies were known to produce fewer males.^[^ ^53^, ^54^ ^]^ This was set to 0.837352526 by default so that the higher biomass and slightly different lifecycle of polygyne colonies did not allow them to be selected at a higher rate.

The number of new queen progeny was generated based on a Poisson distribution and was based on the age of that colony. This was because even though polygyne colonies are larger, the workers are typically smaller and less healthy than those in monogyne colonies. Polygyne colonies might be less healthy per unit of biomass, but the new queens they produce are smaller too. The model thus approximated that both colonies produced the same number of queens at the same age, though drive queens produced proportionally fewer based on their fitness cost. Specifically, it was assumed that 10 000 ants were needed for basic colony maintenance, and that each additional 10 800 ants in the colony produced a single new queen (the actual number of female alates produced in real colonies is larger, but this number was reduced to reduce computational burden while still allowing high variation in the survival of offspring from each colony, even at low density when survival was high).

After the offspring were produced, they could migrate based on a normal distribution to build their own colony around their mother with a standard deviation equal to *average* *dispersal*  ×  √π/2. The position was redrawn if the offspring was placed outside the boundaries of the arena. The default monogyne average dispersal distance was 100 m (0.054159 in SLiM),^[^ ^55^ ^]^ while the dispersal distance for polygyne colonies was set at half of this value due to a lack of sufficient body mass/energy reserves to support sustained flight.^[^ ^56^ ^]^

Age 0 colonies have a survival of:

(1) $$\begin{array}{cc} & \mathrm{Survival}\;\mathrm{rate}\\ & \;=\frac{0.1\times \mathrm{low}\;\mathrm{density}\;\mathrm{growth}\;\mathrm{rate}}{\left(\mathrm{low}\;\mathrm{density}\;\mathrm{growth}\;\mathrm{rate}-1\right)\times \mathrm{competition}\;\mathrm{ratio}+1} \end{array}$$

The biological differences between monogyne and polygyne ant colonies have implications for colony behavior, social dynamics, reproductive strategies, and so on. Polygyne colonies are composed of multiple queens with a shorter lifespan than monogyne queens.^[^ ^39^, ^57^, ^58^ ^]^ Further, newly mated polygyne queens typically join existing polygyne colonies rather than establish their own colony.^[^ ^59^, ^60^ ^]^ To simplify this in the model, polygyne colonies were still represented as a single genotype, but they were allowed to change their genotype more rapidly than the lifespan of a normal polygyne queen, representing the influx of new queens that increasingly dominate offspring production. Thus, each year, all polygyne colonies had a chance to replace their representative reproductive queen genotype, which was set as 50% as default. Colonies unsuccessful in queen replacement experienced colony mortality with a probability of 80%. If they remained viable, the genotype of the previous queen persisted. Further, the greenbeard effect in polygyne colonies, associated with the acceptance of multiple polygyne queens within a nest, results in *SB/SB* genotype new alate queens from polygyne colonies being eliminated by workers.^[^ ^61^ ^]^ The *Sb/Sb* genotype is generally lethal in females,^[^ ^61^ ^]^ so polygyne colonies only produce *SB*/*Sb* (polygyne) queens and tended to have 50% female reproductive individual mortality (the *SB*/*SB* and *Sb*/*Sb* new queens). It was not known exactly what might happen to monogyne colonies with offspring carrying polygyne alleles (due to having a male mate with *Sb*). To make monogyne alleles somewhat competitive (though still at a frequency‐dependent disadvantage, see below) with polygyne alleles, it was assumed that new monogyne queens would only mate with *SB* males, even if their mate was from a polygyne colony.

### 2.3 Colony Mortality

The survival rate of fire ant colonies in this model was influenced by their age and the level of competition they experience from other colonies. Monogyne fire ant queens could live up to 7–8 years.^[^ ^62^ ^]^ Thus, a 50% age‐related death rate of age 6 colonies was implemented, and no colony survived past 7 years in the model. Limited knowledge is available about the lifespan of polygyne colonies, which are characterized by multiple queens and a continual influx of new queens. Thus, it was assumed that they could persist for up to 70 years (chosen because the number of colonies that reach this age was negligible). However, colony lifespan tends to be substnatially less in the presence of a suppression drive (see below).

Following the observed growth rate of new colonies,^[^ ^63^ ^]^ the colony size was increased as it aged, which affected how much competition that colony exerted on others and how much their own mortality rate would be affected by competition. The colony size growth curve was approximately logistical.^[^ ^64^ ^]^ Monogyne colonies followed the equation:

(2) $$S_{monogyne}=\frac{170\;000}{1+66e^{-1.3t}}$$

where *S* is the colony size (in number of ants) and *t* represents the age in years. Polygyne colonies usually contain ≈2 times the number of workers as monogyne colonies of similar age,^[^ ^40^ ^]^ so here the same colony size equation form was used, but the maximum colony size was changed to 340 000:

(3) $$S_{polygyne}=\frac{340\;000}{1+66e^{-1.3t}}$$

Competition experienced by each colony (used for monogyne and polygyne ‐ though polygyne colonies will actually tend to experience less, see below) was calculated as: $Expected\;competition=\sum_{t=2}^{t=7}S_{monogyne_{t}}\times N_{monogyne}\times \pi \times \frac{1}{3}\times r_{t}^{2}$, where *N_monogyne_* represents the total carrying capacities for monogyne‐only populations, with default values of 100 000. *S_t_* means the colony size in *t* year, and *r_t_* represents the competition radius in *t* year separately. The factor of 1/3 comes from the average interaction strength between the colony that received the competition and the colonies that were the source of the competition, which linearly declined with distance. The competition radius of a monogyne colony was based on territory size. Mature colonies had a territory of 100 m^2^, and younger colonies had a proportional territory area based on their biomass relative to the mature colony.^[^ ^65^ ^]^ The radius yielding this territory size was then doubled, to allow colonies with overlapping territory to compete. Polygyne colonies were assumed to have the same territory area as monogyne colonies of the same age.

Fire ant colonies compete for resources and often directly battle.^[^ ^66^ ^]^ Thus, density‐dependent mortality was implemented for all colonies with monogyne colonies having a density‐dependent survival rate of:

(4) $$\mathrm{Survival}\;\mathrm{rate}=0.95-0.6714\times \exp\left(-\frac{2.222}{100000\;}\times \frac{S_{t}}{R}\right)$$

The competition ratio (*R*) in the equation refers to *competition* *ratio*(*R*)  =  *actual* *competition*/*expected* *competition*. Actual competition experienced by each focal colony was determined by the cumulative competitive pressure exerted by neighboring colonies of both social forms (monogyne and polygyne). In spatial models, this competition acting over the course of several years tended to produce colonies that were substantially more dispersed compared to a random distribution. Thus, to maintain desired growth rates and carrying capacity, the number of offspring was multiplied by 0.95, which was empirically determined by simulations to within 1%.

Polygyne colonies had the same survival rates as monogyne, except that the first factor of 0.95 was replaced by 0.9. This is because despite their increased size, multiple polygyne colony queens still have greater total maintenance burdens than a single monogyne queen. Further, polygyne queens tend to be smaller and less healthy (lower survival rates), and some queens only produce sterile alates, reducing colony efficiency.^[^ ^31^, ^32^, ^56^ ^]^ Polygyne colonies exhibit reduced average worker size relative to monogyne colonies (workers average ≈60% the size of a monogyne worker),^[^ ^67^ ^]^ resulting in diminished competitive capacity. To account for this phenotypic variation in the model, a scaling factor of 0.6 was applied to represent the reduced competitive ability. Thus, the higher number of ants in polygyne colonies still allowed them to exert 20% more competition on their neighbors than monogyne colonies of the same age (it was assumed that a colony's competition ability was directly related to its biomass). Additionally, polygyne colonies do not attack other polygyne colonies,^[^ ^68^, ^69^ ^]^ resulting in reduced competition between them. Of course, they still consume resources that were thereby denied to other nearby polygyne colonies, competing indirectly. This was incorporated into the model by implementing an intraspecific competition coefficient of 1/1.2 between polygyne colonies in the competition calculation (the exact resource and energy usage here has not been studied, so the model assumed that larger polygyne colonies exerted the same amount of competition pressure on each other as a smaller monogyne colony of the same age). Overall, this allowed polygyne colonies to slowly outcompete monogyne colonies in the course of a simulation with default parameters in the absence of gene drive intervention (Figure S1, Supporting Information). This appeared to match field observations,^[^ ^31^, ^70^ ^]^ though the greater dispersal ability of monogyne allows it to remain ecologically viable in real‐world settings where additional factors might be present.^[^ ^71^ ^]^

### 2.4 New Drive Variants

Two updated homing suppression strategies were also incorporated into this model. The first was the dominant sterile resistance strategy, in which the presence of a single resistance allele leads to female sterility,^[^ ^46^, ^72^ ^]^ and females lacking a wild‐type allele are also still sterile. Another strategy was the distant‐site/two‐target suppression drive, where the homing drived targets and rescues an essential gene at its own site, with additional gRNAs inducing cleavage at a separate female fertility gene.^[^ ^47^ ^]^ This strategy often has improved genetic load (suppressive power) in diploids compared to standard suppression gene drive (with recessive female‐sterile resistance),^[^ ^72^ ^]^ depending on the exact performance parameters.

Given the shorter generation time of polygyne colonies, novel gene drives associated with this social form were modeled to investigate the impact on suppression gene drive efficacy. Three variants of this homing suppression drive targeting female fertility were considered.

Polygyne drive: This drive contained a copy of the *Sb* gene, which determined the fire ant colony social form. If a monogyne female inherited one copy of the drive, it would develop into a polygyne colony and adhered to all the rules that applied to polygyne colonies (see below). Colonies would remain viable if they were *Sb*/*SB* heterozygotes with a drive.

Greenbeard drive: This drive rescued *SB* homozygous new queens from the greenbeard effect in polygyne colonies (where they were normally removed). They would be able to create new drive monogyne colonies.

Cleave *SB/Sb* drive: In this drive, in addition to its normal functions, it could also cleave the *SB* allele in the germline of polygyne queens, converting it into an *Sb* allele. Thus, all new queens would survive in polygyne colonies where the queen mated with a wild‐type male. However, if the queen mated with a polygyne male, all workers would be *Sb* homozygotes, and the queen would thus be treated as sterile/nonviable. The reverse situation was also modeled, where *Sb* alleles were converted to *SB* alleles in new queens. For both drives, it was assumed that this process happened with ideal efficiency.

### 2.5 Native Ants

To evaluate the impact of native ants on the effectiveness of the homing suppression drive in controlling fire ants, another ant species was introduced. In this model, native ants were represented solely by a single species with monogyne colonies. To simplify the model, it was assumed that monogyne fire ant and native species shared the same life cycle and reproduction rules. In this model variant the competition strength experienced by each colony came not only from its own species but also from the competing species. Hence, the expected competition in each species (both monogyne and monogyne) was calculated as: $Expected\;competition\;for\;fire\;ants=\;\sum_{t=2}^{t=7}S_{monogyne_{t}}\;\times \;N_{monogyne}\;\times \;\frac{1}{3}\;\times \;p_{t}\;\times \;r_{t}^{2}+f\;\times \;\left(\sum_{t=2}^{t=7}S_{native\_t}\;\times \;N_{native}\;\times \;\frac{1}{3}\;\times \;p_{t}\;\times \;r_{t}^{2}\right)$; $Expected\;competition\;for\;native\;ants=f\;\times \;\left(\;\sum_{t=2}^{t=7}S_{monogyne_{t}}\;\times \;N_{monogyne}\;\times \;\frac{1}{3}\;\times \;p_{t}\;\times \;r_{t}^{2}\right)+\sum_{t=2}^{t=7}S_{native\_t}\;\times \;N_{native}\;\times \;\frac{1}{3}\;\times \;p_{t}\;\times \;r_{t}^{2}$, where *f* represents an interspecies competition factor between fire ant species and native ant species with a value between 0 and 1. Thus, each species had reduced interspecific competition compared to intraspecific competition. In the absence of gene drive, both species would thus exist at equilibrium. The default capacity was set at 50 000 for monogyne fire ant colonies, 30 941 for polygyne fire ant colonies, and 50 000 for native ants, with all other parameters remaining consistent with the fire ant‐specific model. Thus, native ants were assumed to reach only half the total territory size that fire ants occupy in the region, with the underlying assumption that they were only able to persist due to the presence of certain microhabitats or ecological niches that do not fully overlap with fire ants.^[^ ^73^ ^]^

### 2.6 Model Scenarios

For most simulations, gene drive males equivalent to 15% of the total males produced by native colonies were released randomly over the entire arena, of which half had the polygyne *Sb* allele. This release was repeated for the first six years of the simulation.

The invasion process of fire ants was also modeled. For these simulations, fire ants were initially limited to the left 40% of the modeling arena, with the left half of this region occupied by polygyne form fire ants at equilibrium density, while monogyne fire ant colonies were similarly placed in the right half of this region. The remaining 60% of the arena was filled by native ants (Figure 8A). These simulations had different parameters for interactions with the competing species (here, assuming that they were being outcompeted and eliminated by fire ants rather than just being confined to a more limited ecological niche). Given the highly invasive nature of fire ant species, the competition factor between the two species was set to 0.5, but in this scenario, this only reduced the competition exerted by native ants on fire ants. In contrast, native ants experienced full competition pressure from fire ants. This represented a scenario in which native ants would eventually be driven to extinction by fire ants in the modeled region. The average dispersal distance in this model was somewhat increased to 138.75 m (the maximum value when varying this parameter) to allow for more rapid fire ant invasion.

Unless otherwise specified, simulations were ended 100 years after the drive release if the population or the drive were not eliminated earlier. Default values for parameters (which were used unless otherwise specified) are shown in **Table** **1**. The default drive parameters were calibrated based on a successfully constructed drive in *Anopheles gambiae* ^[^ ^74^ ^]^ representing an effective drive, but still with several imperfections. Population dynamics were quantified using an ant biomass metric,^[^ ^75^ ^]^ with units defined as the mass of a monogyne colony worker:

(5) $$\begin{array}{c} ant\;biomass=\sum_{age1\;colony}^{mature\;colony}colony\;size*\;\times mass\;factor\times \;*\#colonies \end{array}$$

**Table 1:** Default model parameters.

Parameter	Value
Model time step	1 year
Drive conversion rate	0.95
Germline resistance	0.5
Somatic fitness	0.98
Embryo resistance	0.05
Low‐density growth rate	6
Dispersal distance	100 meters^[^ ^55^ ^]^

Parameter	Monogyne	Polygyne
Capacity	100 000	61 882
Proportion starting territory	0.5	0.5
Competition distance	Varies with age^[^ ^65^ ^]^	Varies with age
Drive release	0.15 of capacity	0.15 of capacity
Mate searching distance	=dispersal^[^ ^55^ ^]^	0.5 × dispersal^[^ ^36^ ^]^
Probability of queen change	0	0.5
Male production rate	1	0.837352526^[^ ^53^, ^54^ ^]^

Given the phenotypic distinctions between social forms, where polygyne colonies exhibit larger colony size but produce smaller workers with reduced fitness compared to monogyne colonies,^[^ ^67^ ^]^ a mass factor of 1 was used for monogyne colonies and 0.6 for polygyne colonies (based on the relative worker size). Native ants were assumed to have the same biomass/colony size in each year as monogyne fire ants. Biomass output was often used as a proxy for the overall effectiveness of drive strategies.

### 2.7 Analysis of Potential Target Genes for Fire Ant Homing Suppression Gene Drive

Protein and nucleotide sequences were obtained from the NCBI database (https://www.ncbi.nlm.nih.gov/). Multiple sequence alignments were performed using the MUSCLE algorithm,^[^ ^76^ ^]^ and the resulting alignments were visualized using the platform Jalview version 2,^[^ ^77^ ^]^ gRNA target sites were identified using the online tool CHOPCHOP (http://chopchop.cbu.uib.no).

### 2.8 Data Generation

All simulations were conducted on the High‐Performance Computing Platform of the Center for Life Science at Peking University. Python was used for analyzing data and preparing the figures. All models and data can be accessed on GitHub (https://github.com/jchamper/Fire‐Ant‐Suppression‐Model).

## 3 Results

### 3.1 Suppression Gene Drive in Fire Ants

**Figure 2:**
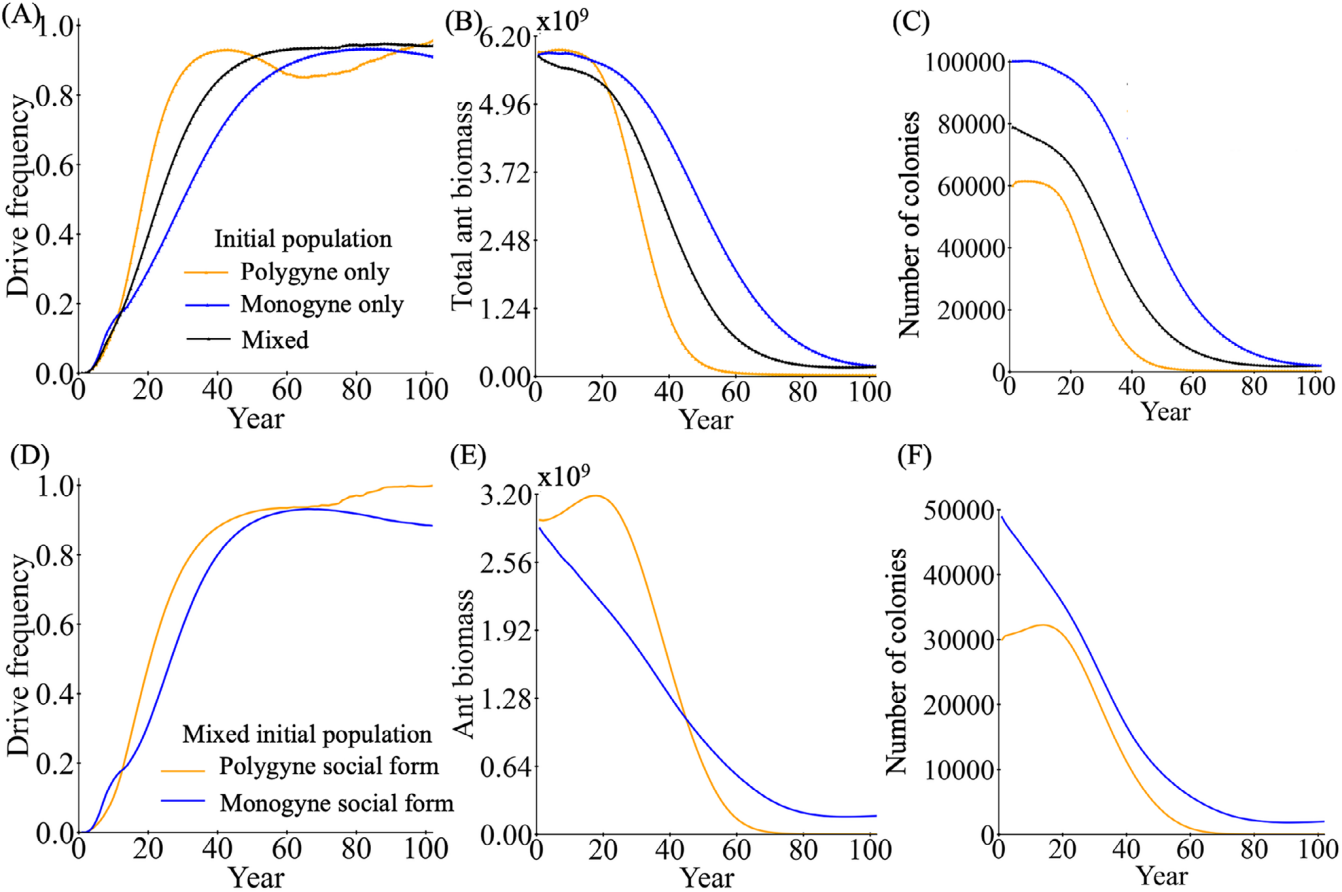
Homing suppression gene drive in fire ant populations. Drive males were introduced into the spatial fire ant population. We examined fire ant populations that were composed only of monogyne colonies, only polygyne colonies, or a mix of both, with each occupying 50% of the initial territory. We display the A) drive frequency, B) total fire ant biomass (in units equivalent to the size of an average monogyne colony worker), and C) number of colonies in each model. For the mixed population, we also separately track the D) Drive frequency, E) ant biomass, and F) number of colonies for each social form. Each line shows the average of 200 simulations.

To evaluate the effectiveness of a suppression gene drive in fire ants, we evaluated a homing suppression gene drive in our spatial model. Fire ant default parameters are based on estimates and field studies (see methods). We first examined the drive dynamics in a monogyne population, a polygyne population, and a mixed population (**Figure** **2**). The drive frequency increased in all scenarios, indicating successful propagation of the drive (Figure 2A). Following the drive introduction, both ant biomass (number of monogyne workers or equivalent mass, Figure 2B) and colony abundance (Figure 2C) declined. While polygyne colonies exhibited complete elimination, monogyne colonies persisted by the end of the simulation (Figure 2B,C). This was most likely mainly due to the difference in generation time (Figure S2, Supporting Information). In a hypothetical panmictic fire ant population at equilibrium, the generation time of monogyne colonies in our model is 5.4 years. For polygyne colonies, with 50% representative queen replacement per year (due to higher individual queen mortality), the generation time is expected to be slightly higher than 2 years, and the polygyne drive allele frequency matched the monogyne trajectory if we assumed their generation time to be 2.3 years (Figure S2, Supporting Information). Though spatial and density‐related factors could influence these results, polygyne ants should in general still have a substantially shorter generation time, accounting for most of the difference in suppression time.

We also examined individual monogyne and polygyne trajectories in a mixed population. Though polygyne can outcompete monogyne without gene drive, the polygyne population was still eliminated more quickly than the monogyne population, even though monogyne experienced a more rapid initial decline (Figure 2D–F). Persistence of monogyne colonies at the very end of the simulation (despite the slower population reduction) for the mixed population was likely due to a chasing effect. This effect occurs when a suppression drive fails to eliminate the target population in a spatial model, despite theoretically having enough suppression power, because wild‐type alleles temporarily escape from the drive by moving into an empty region. In our case, the drive with default parameters generated a genetic load of 0.87, which should have been adequate to eliminate the population given a low‐density growth rate of 6 (which requires a genetic load of 0.83 in a deterministic model).

Due to the long generation time of fire ants, increased importance should be placed on larger initial release sizes compared to other gene drive applications. Varying the release size, we found that modest benefits can be obtained with larger releases (Figure S3, Supporting Information), though these decrease rapidly much past our default release rate of 15% males for six years.

### 3.2 Evaluating the Effect of Drive Performance and Ecology on Population Suppression

**Figure 3:**
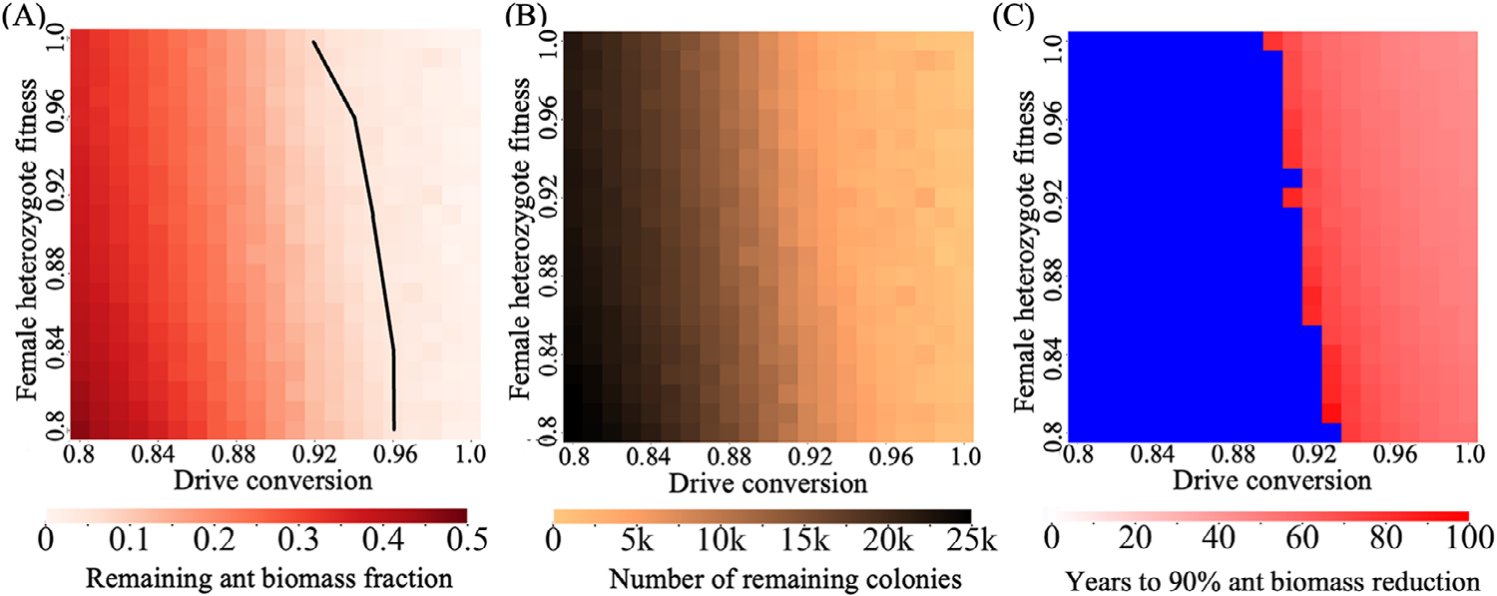
Impact of varying drive efficiency and somatic fitness on fire ant control. The drive, with varying drive conversion and female fitness, was released into a mixed spatial fire ant population. Heatmaps show the A) fraction of remaining fire ant biomass compared to the initial biomass after 100 years (in units equivalent to the size of an average monogyne colony worker), B) the number of colonies remaining in the arena 100 years after the release of drive males (the initial number is 80941), and C) time required to achieve a 90% reduction in fire ant biomass. Blue indicates that the population fails to reach a 90% reduction within 100 years after drive release. The black line in panel A (and area to the right of the line) shows where the drive has a sufficient theoretical genetic load value at equilibrium to eliminate a panmictic deterministic population. Each point shows the average result from 20 simulations (including only simulations that reached 90% biomass reduction).

We next evaluated how drive performance affects the suppression outcome. In this analysis, we varied the drive conversion and female fitness parameters from 0.8 to 1.0, representing a theoretically efficient drive capable of suppressing generic haplodiploid panmictic populations over most of the range.^[^ ^30^ ^]^ Under a perfect drive scenario, nearly the entire fire ant population could be eradicated (**Figure** **3**A,B). Even when fitness and drive conversion rates were reduced, the drive maintained a significant impact, decreasing the total ant biomass by more than 50% (Figure 3A). However, even with a perfect drive, achieving a 90% reduction in ant biomass required ~30 years (Figure 3C). Notably, the impact of reduced somatic fitness was minimal when the drive conversion rate was sufficiently high, underscoring the importance of drive efficiency in determining suppression outcomes.

**Figure 4:**
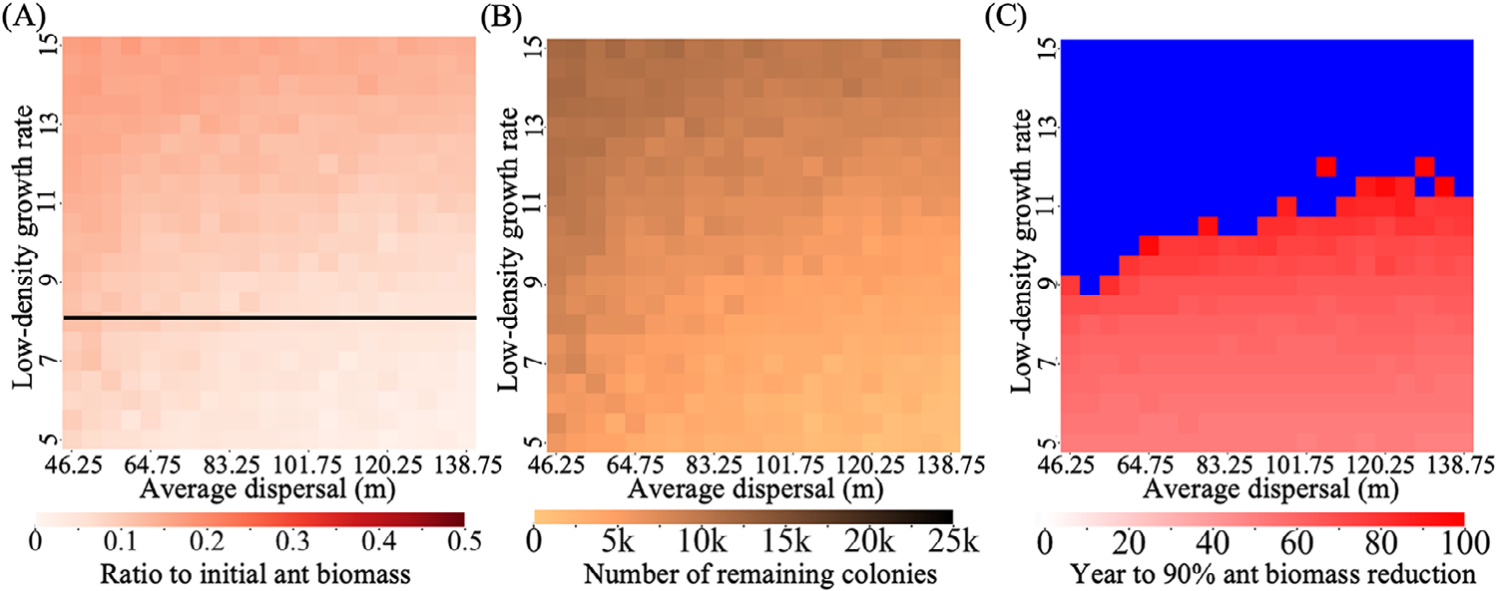
Impact of varying dispersal distance and low‐density growth rate on fire ant control. The drive was released into a mixed spatial fire ant population with varying average dispersal rate (migration distance) and low‐density growth rate. Heatmaps show the A) fraction of remaining fire ant biomass compared to the initial biomass after 100 years (in units equivalent to the size of an average monogyne colony worker), B) the number of colonies remaining in the arena 100 years after the release of drive males (the initial number is 80941), and C) time required to achieve a 90% reduction in fire ant biomass. Blue indicates that the population fails to reach a 90% reduction within 100 years after drive release. The black line in panel A (and the area below the line) shows where the drive has a sufficient theoretical genetic load value at equilibrium to eliminate a panmictic deterministic population. Each point shows the average result from 20 simulations (including only simulations that reached 90% biomass reduction). m ‐ meter.

The habitat occupied by fire ants and the extent of their dispersal may significantly influence the effectiveness of control efforts. To investigate these factors, we varied the growth rate of fire ants in low‐density regions and their average dispersal distance (**Figure** **4**A–C). Our findings indicate that increased dispersal distance can contribute to a subtle reduction in the fire ant population. However, its impact remains minimal when the low‐density growth rate is sufficiently high. This may be because higher dispersal tends to inhibit chasing. Specifically, it involves wild‐type individuals escaping to empty space and gaining reproductive advantages due to reduced competition. Even though the gene drive remains present, population elimination can be indefinitely delayed. Such chasing has occurred in many spatial models of suppression gene drive.^[^ ^28^, ^30^, ^78^, ^79^ ^]^ Chasing may play a role in late‐stage population suppression in our fire ant model (see below). Of course, chasing only tends to be significant when the genetic load (suppressive power) of the drive is theoretically capable of eliminating the target population.

In contrast, a reduced low‐density growth rate contributed to more effective suppression, particularly given that our drive with default parameters lacked the power to theoretically suppress populations with low‐density growth rates much higher than 8. Nonetheless, all simulations within the given parameter range consistently achieved at least a 70% reduction in total ant biomass (Figure 3A), demonstrating that the default drive's performance is highly effective in substantially suppressing fire ant populations, even with possible variations in fire ant ecology.

In polygyne colonies, newly emerged alates typically integrate into existing colonies through ambulatory dispersal rather than establishing independent colonies. This social structure allows for dynamic reproductive queen replacement within the polygyne social form. Our default values for polygyne‐related parameters in the model, including the representative queen replacement rate of the relative dispersal ability, were based on approximate estimates. We thus varied each of these to see how it would impact model outcomes. While polygyne queen dispersal distance minimally impacted population suppression (Figure S4A–C, Supporting Information), it would likely have slowed down the drive wave of advance if the drive release wasn't widespread. Our findings revealed a more nuanced relationship between queen replacement probability and population dynamics. At 30% yearly representative queen replacement probability, population suppression appears most effective (Figure S4D–F, Supporting Information), perhaps because the drive in polygyne won't far outstrip monogyne (due to increased generation times for polygyne), reducing chasing dynamics near the end of the simulation. However, when queen replacement probability is lower, monogyne is now reduced more quickly due to shorter generation time, though this range is unlikely to be realistic (it would require low levels of actual queen replacement and polygyne queens that live longer than monogyne, when the reverse is true).

### 3.3 Chasing in Fire Ants

In our previous simulations, we calculated the necessary genetic load threshold (black line) to eliminate a deterministic population (Figures 3, 4). Population elimination still did not occur in these simulations, so chasing is one possible reason for this. However, with the longer time scale of fire ant suppression, the effect of chasing may be less clear since it takes many generations to fully develop. To better assess this, we first examined the performance of our default drive in a panmictic model compared to our spatial model (Figure S5, Supporting Information). Although initial population reduction rates parallel those observed in the panmictic model, continuous space allows for a higher long‐term population size.

We thus extended the time scale of our spatial model to examine chasing in more detail. We found that for default parameters (where genetic load is sufficient for deterministic population elimination), the total biomass does indeed fluctuate, characteristic of chasing (Figure S6A, Supporting Information), though population elimination did occur in a few replicates. Further, the average nearest neighbor index also fluctuated (Figure S6B, Supporting Information), which shows varying patchiness in the population distribution and is strongly correlated with chasing.^[^ ^80^ ^]^ Visual inspection of fire ant simulation movies also showed chasing behavior over long time scales (https://rb.gy/wnt7fn). Thus, chasing is prevalent in our fire ant models, even though it is not likely to have as large of an impact as in other models where more generations elapse in a shorter time.

### 3.4 Suppression Drive Designs with Higher Genetic Load

**Figure 5:**
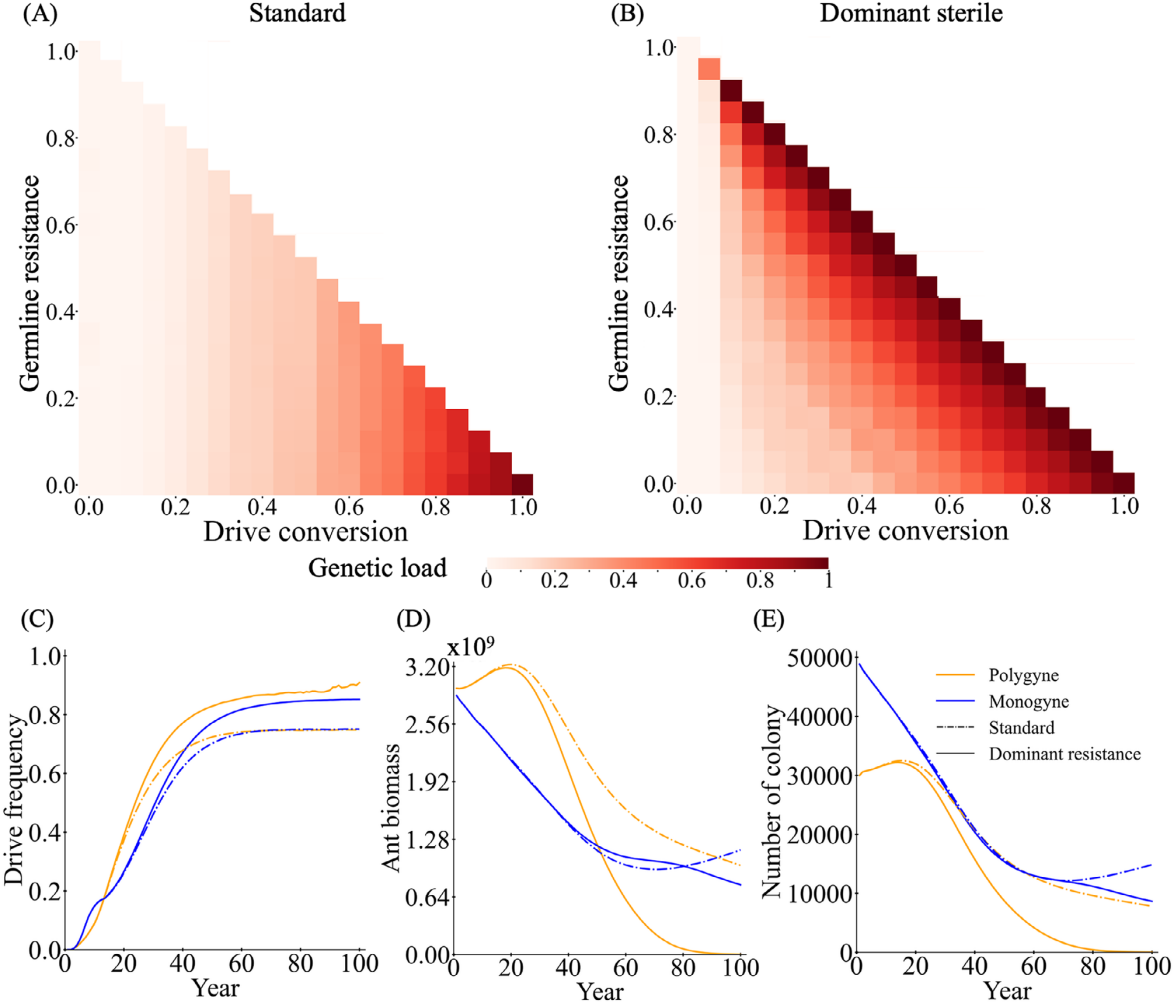
Performance of drives with dominant sterile resistance alleles. Using a generic discrete‐generation panmictic model, the equilibrium genetic load (suppressive power) is shown with default performance characteristics under varying drive conversion rate and germline resistance for A) standard haplodiploid suppression drives targeting female fertility and B) drives that produce dominant‐sterile resistance alleles. 50 simulations were assessed for each point in the parameter range. In the fire ant model with a spatial population (composed of a mix of monogyne and polygyne colonies), a drive conversion rate of 0.8, and a germline resistance allele formation rate of 0.2, we track the C) drive frequency, D) ant biomass (in units equivalent to the size of an average monogyne colony worker), and E) the number of colonies for each social form in simulations with standard and dominant‐sterile resistance suppression drives. Displayed data are the average of 200 simulations.

Given the challenges in constructing standard homing drives with high efficiency by targeting recessive female‐specific fertility genes, we examine alternative approaches to enhance suppression efficiency. Dominant‐sterile homing suppression drives that cause dominant female‐sterile resistance have demonstrated higher efficiency for self‐limiting systems in modeling and in *Drosophila*,^[^ ^46^ ^]^ but these have not yet been evaluated in haplodiploid species or in self‐sustaining drives when the drive remains recessive sterile, which is highly plausible at such target sites. Here, we evaluate the genetic load of such systems, a characteristic used to measure suppressive power on a target population. In a simple discrete‐generation haplodiploid model, we see that for a homing suppression drive with recessive sterile resistance, high genetic load is only obtained when drive conversion is high (**Figure** **5**A). However, for the dominant‐sterile resistance version, high genetic load could be achieved even with low drive conversion, as long as the total cut rate (drive conversion + germline resistance) remains high (Figure 5B). This result is similar to two‐target suppression drives in diploids.^[^ ^47^ ^]^

While dominant‐sterile resistance yields higher genetic load in the long term, the drive conversion is still the main factor in how rapidly the drive frequency increases at first, and time to population elimination is a critical consideration in fire ants. In species‐specific modeling for fire ants with a less efficient drive, we nonetheless found that the dominant female‐sterile resistance system demonstrates superior suppression efficacy compared to the standard drive within a few decades (Figure 5C–E), with this difference particularly pronounced in polygyne versus monogyne social forms. The dominant female‐sterile resistance system exhibits comparable release ratio‐dependent dynamics to those observed in the standard homing drive. Increased release ratios expedite suppression across both default drives and even drives with higher fitness costs (Figure S7, Supporting Information).

A distant‐site/two‐target homing suppression drive design^[^ ^47^ ^]^ demonstrates similarly improved genetic load based on total cut rate in haplodiploids (Figure S8, Supporting Information). This system involves a homing rescue drive (usually considered for population modification) that has additional gRNAs targeting a female fertility gene (the same type as found in our normal homing suppression drive and specifically not a site that produces dominant‐sterile resistance alleles). The two‐target site design has two possible configurations, which represent a tradeoff. If the drive site gene is essential but haplosufficient, then 100% cutting will reduce genetic load, with an optimum at a somewhat lower level of total cutting (Figure S8C, Supporting Information). Under high cutting rate conditions, the accumulation of nonfunctional resistance alleles at the drive site attenuates drive establishment, thereby slowing down the disruption of the female fertility gene and consequently reducing genetic load. If the drive site is at a haplolethal gene, which is a more difficult target for rescue, then genetic load continues to increase as the total cut rate increases (Figure S8D, Supporting Information), comparable to dominant female‐sterile resistance. Despite equivalent equilibrium genetic load at 100% cutting, the haplolethal distant‐site drive system has somewhat higher genetic load when performance is less ideal (Figure S8, Supporting Information). Further, population suppression compared to dominant female‐sterile resistance is somewhat faster (Figure S9, Supporting Information).

### 3.5 Strategies to Enhance Suppression Effectiveness by Manipulating Social Form

Building on previous results, which demonstrate that the polygyne social form is more amenable to control, we propose that converting the social structure of fire ants could represent a promising strategy for enhancing suppression efforts. Actual implementation of such methods would require a more detailed understanding of the molecular mechanism of social form, but such investigations could be readily conducted if economical genetic modification techniques are developed for fire ants in the future.

**Figure 6:**
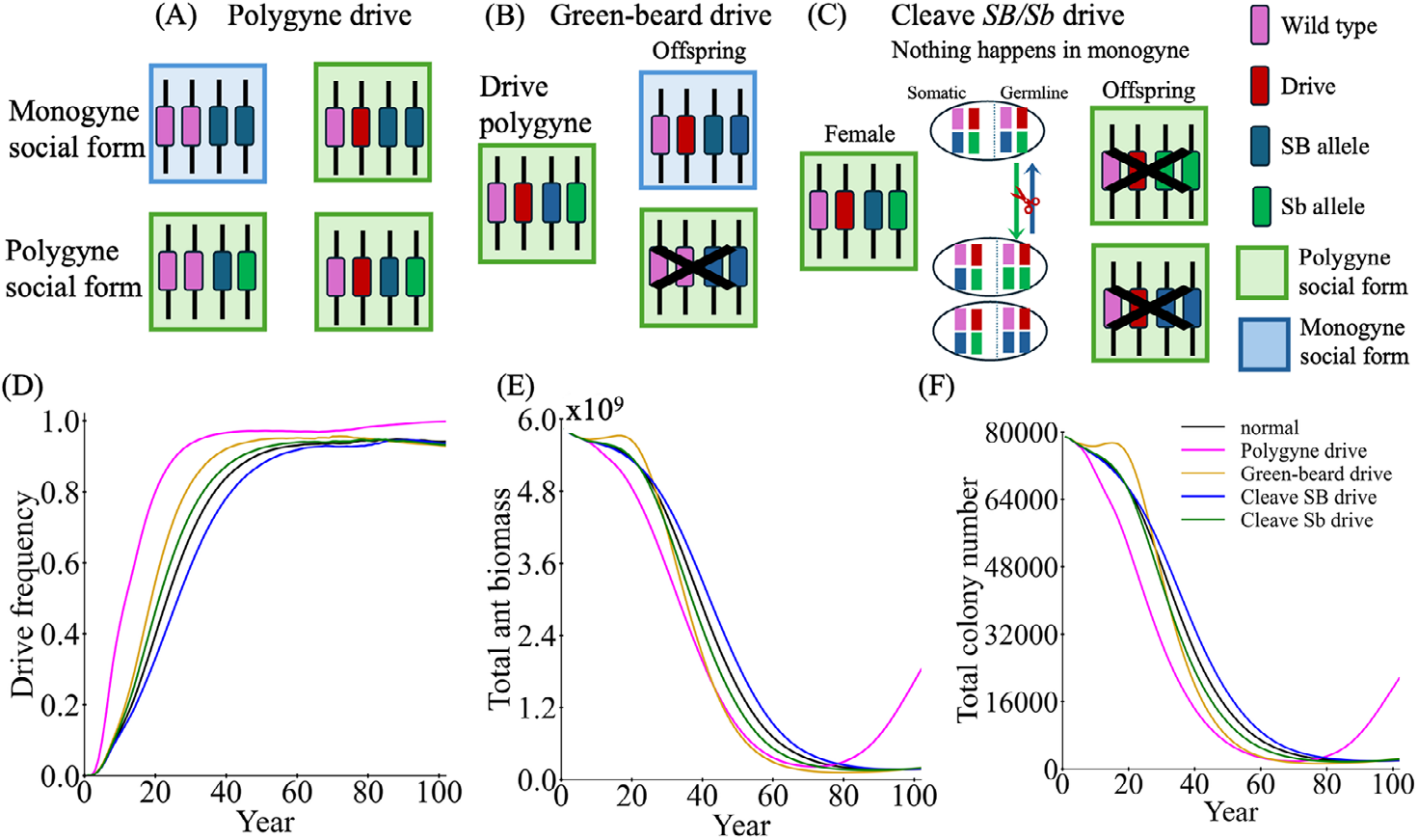
Comparison of new drive variants. A) In polygyne drive, all drive queens will be polygyne, even if their genotype is *SB*/*SB*. Queens are still viable if they have at least one *SB* allele at the native polygyne locus. B) In greenbeard drive, the *Sb*/*Sb* new queens born in polygyne colonies will remain viable if they have a copy of the drive. C) In the cleave *SB/Sb* drive, the *SB* allele or *Sb* allele is cut in the germline of drive carrying *SB*/*Sb* heterozygotes, converting it to a *Sb* or *SB* allele, which then is inherited by all offspring. Each drive was released into a mixed spatial fire ant population, and the D) drive frequency, E) ant biomass (in units equivalent to the size of an average monogyne colony worker), and F) number of colonies were tracked. Displayed data are the average of 200 simulations.

Three strategies were designed to enhance the control of fire ants. One strategy involves converting all monogyne colonies into a polygyne social form. The drive contains a dominant element that ensures that drive carriers will have a polygyne colony form, even if they lack an *Sb* allele (**Figure** **6**A). Sb carriers with a drive remain viable if they still have an *SB* allele. This approach would shift the population toward the polygyne social form, leading to a significant reduction in the number of monogyne colonies. Another approach involves a drive engineered to counteract the “greenbeard” effect by rescuing SB homozygotes in polygyne colonies if they carry a drive, thereby increasing the number of drive offspring in polygyne colonies that can lead to a more rapid drive increase in the monogyne population (Figure 6B). A final approach involves targeting the *SB* or *Sb* allele for cleavage by the drive allele, promoting conversion to the alternate *Sb* or *SB* allele in germline cells that have a drive and are also *SB/Sb* heterozygotes (Figure 6C). When converting *SB* to *Sb* alleles, polygyne colonies may have more offspring if the queen is mated to an SB male, but if mated to an Sb male, the queen will not be viable because her offspring will all be Sb homozygotes (she will essentially be sterile, resulting in colony nonviability). While monogyne colonies tend to produce more males (and wild‐type polygyne colonies produce half SB males), this drive will be at a disadvantage when it reaches high frequency due to increased fractions of *Sb* alleles in the population. Alternatively, converting *Sb* to *SB* alleles will prevent queens from generating new queen progeny when mated to an SB male (workers from other queens in the colony would eliminate her SB homozygous queens, and we thus don't allow her to be the representative queen in the colony), but this approach may still be better because it allows for the drive frequency in monogyne colonies to increase more rapidly, and monogyne ants are often more of a limiting factor in population suppression.

Modeling these results, we found that the polygyne drive results in a population resurgence around the 70‐year mark for both social forms (Figure 6D–F; Figure S10, Supporting Information). Though this drive increases most quickly at first, it lacks the ability to persist in monogyne, which eventually can recolonize empty space. The greenbeard mechanism shows an initial minor increase in monogyne colonies, but then demonstrates an enhanced suppression rate compared to the normal drive system, especially for the polygyne form (Figure 6D–F; Figure S10, Supporting Information). Comparative analysis of the cleave SB drive reveals that this construct has slightly inferior efficiency relative to a standard suppression drive, but the version cleaving Sb offers somewhat superior performance (Figure 6D–F; Figure S10, Supporting Information).

### 3.6 Competing Species in the Fire Ant Model

**Figure 7:**
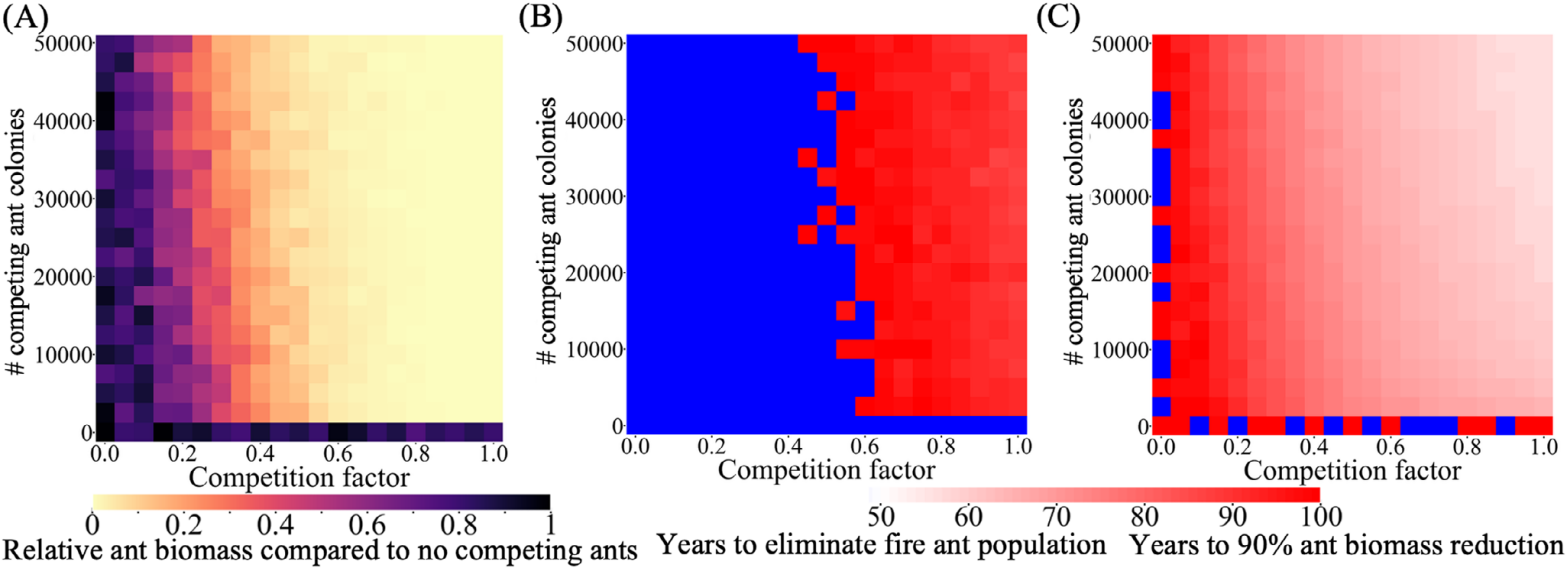
Gene drive suppression of fire ants is boosted by a competing species. The drive with default parameters (except for the low‐density growth rate, which was increased to 10) was released into a spatial mixed fire ant population. The interspecific competition factor and the equilibrium number of competing ant colonies were varied. Heatmaps show A) the relative fire ant biomass at year 100 after drive release (compared to identical simulations without competing ants) (in units equivalent to the size of an average monogyne colony worker), B) the time to population elimination following drive release (usually close to 100 years), and C) the time to achieve a 90% reduction in fire ant biomass, with persistence beyond 100 years indicated in blue. Displayed data are the average of 20 simulations (including only simulations with population elimination).

Our previous research revealed that competing mosquito species could aid a gene drive, allowing stronger suppression and avoiding chasing.^[^ ^44^ ^]^ As a highly invasive species, understanding the mechanisms by which fire ants invade and establish colonies in new regions, as well as how native ant populations respond to their presence, is crucial. With ants present in high numbers in almost all biomes, any invasion of fire ants is likely to be at least somewhat opposed by the native ant species, some of which may persist in a reduced ecological niche even if the fire ant invasion is highly successful. To examine the impact of such native ants on gene drive outcomes, we simulated scenarios where fire ants and native ants coexist (though with a higher starting fire ant population), with symmetrical competition factors, representing the relative competition strength between the two species. We found that the presence of native ants contributes substantially to the suppression of the fire ant population, facilitating successful suppression with greater competition (**Figure** **7**A,B). In fact, complete fire ant elimination becomes possible, though this still requires a temporal window exceeding 80 years (Figure 7C). This timeline suggests the necessity for long‐term management strategies despite the beneficial effects of native ant competitors. On the other hand, it also suggested that good ecosystem management maximizing the health of native species, may allow greater success in gene drive deployments against invasives.

### 3.7 Drive Deployment to Halt a Fire Ant Invasion

**Figure 8:**
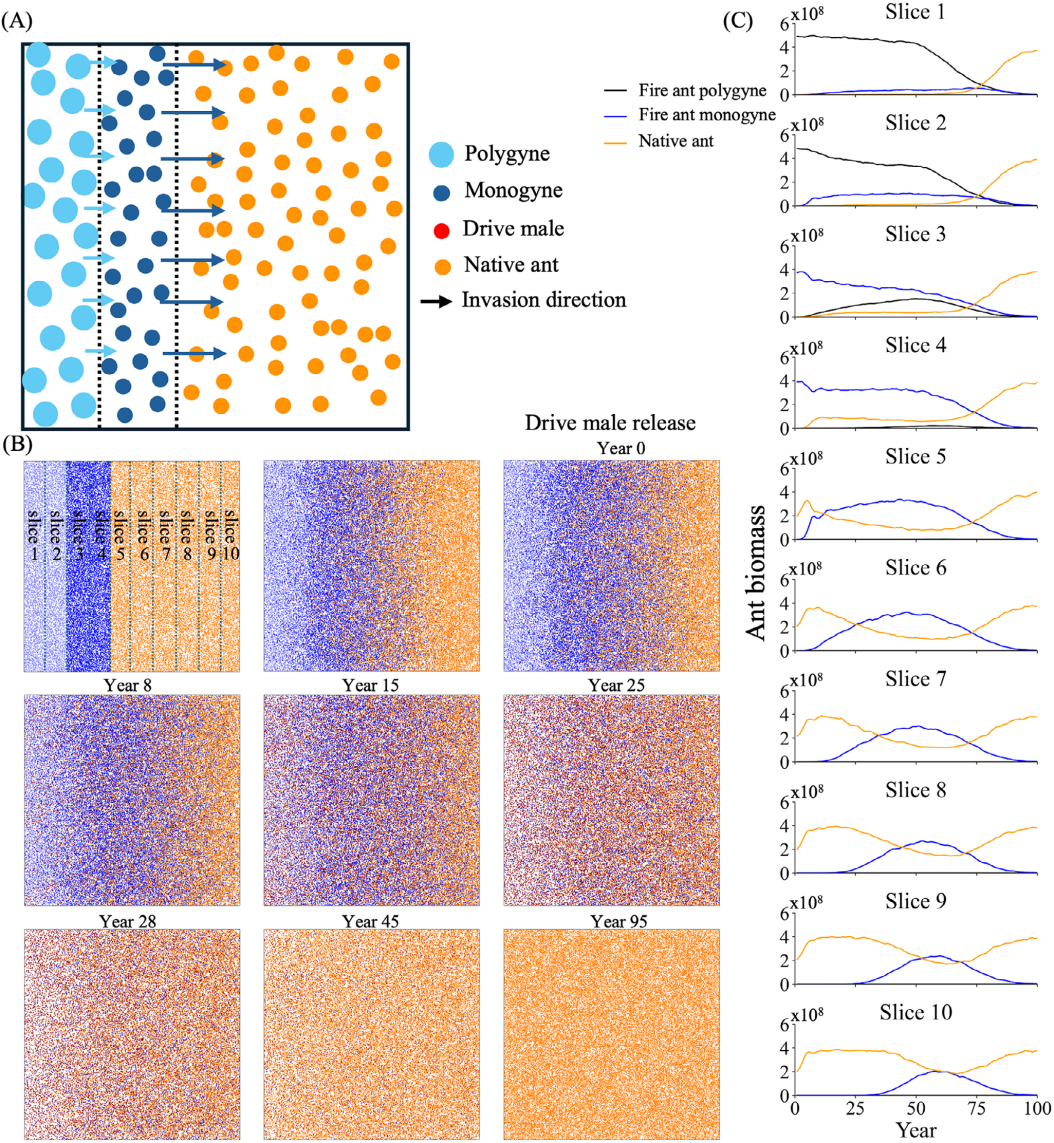
Gene drive against an invading fire ant population. A) A diagram of the simulation start and invasion process. Red gene drive individuals are released in the population when the biomass in the left half of slice 7 is composed of 50% fire ants. The arrows indicate the invasion speed, which is proportional to the average dispersal. Native ants suffer full competition from fire ants, but their competition on fire ants is reduced by half. B) Snapshots of the invasion process. The blue dashed lines indicate 10 slices used for tracking ant biomass. C) The ant biomass (in units equivalent to the size of an average monogyne colony worker) of each 10% slice for native ants and each social form of fire ants. Year 0 is the year of the drive release.

In some cases, native ants may not have a unique ecological niche to allow them to partially persist in the face of a fire ant invasion. They will then tend to only coexist with fire ants for limited time scales on the leading edge of an invasion. Here, we started a scenario where such weaker native ants are being pushed back by fire ants, with slower dispersing polygyne fire ants following behind monogyne (**Figure** **8**A). Drive release was initiated when fire ant populations had partially advanced into the native population after the start of the simulation, allowing us to evaluate the efficacy of gene drive intervention. Monogyne colonies were the primary contributors to the high invasive strength of fire ants due to their rapid territorial expansion through nuptial flights. They swiftly advanced, but eventually lost their competitive advantage when the drive reached high frequency, retreating and rapidly declining in frequency (Figure 8B). In contrast, polygyne colonies exhibited more limited dispersal and were also eliminated more quickly by the drive. Through the implementation of a suppression gene drive, native ant populations were able to reclaim the entire territory, even in regions that had previously been completely colonized by fire ants, though the drive needed to progress for a few decades before native ants began to recover (Figure 8C).

### 3.8 Identification of Female Fertility Target Genes in Fire Ants

Due to the difficulty in generating constructs in fire ants, even assuming steady future advances in genetic engineering technology, it will be important to use related gene drive studies to more rapidly generate viable gene drives. We thus considered several female fertility target genes in fire ants as potential candidates, desiring highly conserved regions, sites for implementing a multiplexed gRNA strategy, and previous demonstrations of suppression drives targeting homologs in other insects.^[^ ^81^ ^]^ Potential target genes included the female‐specific exon of *doublesex*, *intersex*, *virilizer*, *Octopamine β2 receptor*, *NADPH oxidase*, *stall*, and *nudel*. Multiple sequence alignment was performed across species including *Solenopsis invicta*, *Linepithema humile* (invasive Argentine ants), *Aedes aegypti*, *Anopheles stephensi*, *Drosophila melanogaster*, *Vespula vulgaris* (invasive wasps), *Bemisia tabaci* (the whitefly, an agricultural pest), and *Spodoptera frugiperda* (the fall armyworm, also an agricultural pest).

Two of these genes, *stall* and *nudel*, failed to exhibit substantial regions of conserved sequence (Figure S11, Supporting Information). The female‐specific exon of *doublesex* also exhibited poor nucleotide‐level conservation, in contrast to its high conservation between many other species.^[^ ^24^ ^]^ Therefore, only the fire ant ortholog of this sex‐specific exon was directly employed for gRNA design, targeting early in the exon to potentially generate dominant‐sterile resistance alleles.^[^ ^46^ ^]^ Highly conserved regions of the remaining four genes were selected for the design of gRNA target sequences. Such regions that are strongly associated with evolutionary conservation across species tend to have high functional significance. Thus, targeting them likely favors the formation of nonfunctional resistance alleles instead of functional resistance. Nucleotide sequences of conserved regions in fire ants were subsequently identified and used for guide RNA target site selection and design (Figure S12, Supporting Information). These were designed for maximum multiplexing efficiency, placing target sites as close together as possible while still keeping to a low level the chance that a mutation at one gRNA target site will block another gRNA from binding.

## 4 Discussion

The fire ant is an exceptionally challenging invasive species to control. In this study, we systematically investigated the potential of suppression gene drive as a novel fire ant management strategy. Our analysis demonstrates the potential efficacy of suppression gene drive in managing fire ant populations, albeit with a substantially extended time scale compared to other gene drive applications due to the fire ant's relatively long generation time for an insect.

The propagation dynamics of homing drive are significantly influenced by the reproductive strategies of the target species.^[^ ^45^, ^82^ ^]^ In fire ants, queens engage in a single mating event and retain sperm for the reminder of their reproductive lives without remating.^[^ ^33^, ^54^, ^83^ ^]^ If these are sterile, they will be nonviable, and fire ant colonies compete with their neighbors for resources. Likely because of these reasons, we found that overall suppression efficiency for fire ants was worse than for generic haplodiploids.^[^ ^30^ ^]^ Further, the long generation time slows suppression gene drive, but it also potentially makes it easier to achieve high release rates, since several yearly releases can take place within a single generation. Such high releases would be useful for achieving suppression on reasonable time scales, and rearing fire ants in the lab can be performed readily. To enable releases of males during the mating season, though, it is possible that semi‐field rearing strategies would be required so that released drive individuals perform their nuptial flight at an appropriate time. Moreover, even the release of new queens would not result in an initial increase in ant biomass, which is advantageous as it eliminates the need for sex sorting and monitoring the precise timing of mating seasons (semi‐field colonies at a release site could be allowed to reproduce naturally). Matings between polygyne queens and released drive males or even direct release of polygyne queens may be particularly valuable because polygyne queens could join established nests, allowing them to produce drive alates more quickly.

Our simulations demonstrated that polygyne populations exhibited increased susceptibility to suppression, indicating that social structure composition substantially influences drive system efficacy. This dynamic is most likely due to their shorter generation time of polygyne queens, which live shorter and share the nest with other queens that may be continually arriving each year.^[^ ^84^ ^]^ In territories dominated exclusively by polygyne social forms, our model predicts complete population elimination, resulting in successful habitat restoration (Figure 2). Targeted strategies can exploit this vulnerability to facilitate suppression in mixed populations, where generation times do not directly predict gene drive performance due to intermating between the social forms. In some drive systems designed to induce social form transformation, population suppression efficiency could be enhanced. Indeed, such greenbeard mechanisms are prevalent among eusocial insects and are often vulnerable to “false beard” cheating, where some individuals lacking the greenbeard trait use a different mechanism to obtain better treatment from greenbeard individuals.^[^ ^85^ ^]^ By exploiting this phenomenon, we tested a system that manipulates the greenbeard mechanism, allowing monogyne new queens with the drive to survive greenbeard effects in polygyne colonies, enhancing suppression efficiency by allowing polygyne colonies to more heavily contribute to drive frequency in monogyne populations, which are the limiting factor for population suppression. These drive designs may currently be difficult to implement in practice, though they may become possible, together with other novel alternatives, as more studies are conducted on the mechanism behind social form behavior in fire ants and related species. For example, it might be possible to design transgenes to allow monogyne drive colonies to escape from aggressive interactions with polygyne colonies.

To further enhance suppression outcomes, two additional improved drives (dominant‐sterile resistance and two‐target/distant‐site drives) were developed based on the original homing suppression drive. Although careful design is required to achieve dominant‐sterile resistance,^[^ ^46^ ^]^ it has the potential to achieve a higher genetic load compared to other drive systems with the same parameter set. The two‐target design offers similar improvements but requires additional gRNAs compared to a standard drive.^[^ ^47^ ^]^

While our model incorporates a standard dispersal parameter, nuptial flight distances in fire ants exhibit considerable individual variation, with documented dispersal ranges demonstrating significant heterogeneity during reproductive flights.^[^ ^55^ ^]^ However, our results demonstrated that with widespread drive release (likely necessary for drive success on acceptable time scales) the nuptial flight distance exerts minimal influence on suppression outcomes, though dispersal likely assumes greater ecological significance during invasions, and mostly likely would affect long‐term chasing dynamics in fire ants too if they manage to persist in the face of the drive and other pressures (Figure 4).

Biological invasion represents a complex process, with ecological constraints and selective pressures.^[^ ^86^, ^87^ ^]^ The release of gene drive during the invasion interferes with its progression, facilitating the elimination of fire ants and recovery of native ants (Figure 8). Indeed, interspecific competition may play a more substantial role in regulating fire ant populations within shared ecosystems (Figure 7). These dynamics increase the chance of successful suppression outcomes.^[^ ^44^ ^]^ Even if fire ants tend to be dominant, native ants can provide at least some significant competition,^[^ ^88^, ^89^ ^]^ and models must incorporate this process. Specifically, studies have shown that fire ant territory size is highly modulated by interspecific competitive interactions, especially the relative agonistic capacities of adjacent heterospecific colonies.^[^ ^90^ ^]^ The presence of these native species can reduce colony establishment success in fire ants by increasing the likelihood of brood raiding during colony foundation and incipient stages,^[^ ^91^ ^]^ just as in our model.

Despite incorporating multiple ecological factors, our model has limitations regarding niche‐specific demographic and geographic variables that may influence drive suppression outcomes. Landscape heterogeneity may influence fire ant transmission dynamics and release patterns. Uncertainty in understanding interactions between social forms across different regions could also affect ultimate outcomes. We also assumed long‐term persistence of polygyne colonies, but ant habitats may be more widely variable, with natural extinction‐recolonization cycles that may facilitate gene drive suppression by reducing effective generation times. Many of our model parameters were also estimated based on a limited number of studies (Table 1). Future research could investigate these factors in more detail, allowing a better understanding of fire ant control methods while also providing fundamental knowledge about various aspects of this interesting species. Modeling could also potentially be used to investigate the mechanisms of monogyne and polygyne coexistence by adding additional features and examining parameter ranges where their population dynamics more precisely match ecological field studies.

To actually deploy gene drives in fire ants or other invasive ants such as Argentine ants or crazy ants, additional advances will be needed in ant genetic engineering. However, long‐term prospects are promising in this field. Genetic knock‐outs have been achieved in fire ants,^[^ ^92^ ^]^ and knock‐ins in another ant species.^[^ ^93^ ^]^ One major limitation is rearing ants in the lab that produce alates at appropriate times. Additional research may overcome this, but more resource‐intensive semi‐field/outdoor conditions may be needed to ensure full lifecycles among research subjects. Such considerations also apply to actual releases of fire ants. One possible way of overcoming this may be to rear several colonies in the lab and then release them after a few years, avoiding early colony mortality, and allowed each “released” colony to produce many alates over the course of a few years. A pest control program conducted immediately prior to fire ant release would also allow released individuals to represent a greater fraction of the population, substantially accelerating suppression (given the long generation time, this consideration would be more important than in other species) or reducing release requirements. This strategy could be further boosted by co‐releasing wild‐type native ants if they have been displaced from the region by fire ants and would otherwise take longer to return.

Overall, we conclude that gene drive in fire ants is promising but likely to be a highly challenging application, both due to the difficulty in generating the needed ants and releasing them at large scale. Even then, time scales for high population suppression will optimistically still extend over a few decades. Though long compared to many other gene drive applications, there is precedent for long genetic pest control programs, such as those against the New World Screwworm^[^ ^94^, ^95^ ^]^ or *Aedes aegypti* mosquitoes.^[^ ^96^ ^]^ Many pesticide‐based control programs are indefinite in nature, and for a gene drive in fire ants, the “active” part of the program requiring releases and heavy investment would be far shorter than the total program duration. This still means that other short‐term fire ant control research remains essential. However, long‐term outcomes for gene drive appear to be positive, particularly with improved variants, and other methods of control may be less efficient and more resource‐intensive, even if they yield more immediate benefits. Thus, long‐term investment in genetic and rearing technique research could yield major long‐term benefits for gene drive‐based control in fire ants and potentially other major invasive ant species that have similar population dynamics.

## Conflict of Interest

The authors declare no conflict of interest.

## Supporting information



Supporting Information

## Data Availability

All models and data can be accessed on GitHub (https://github.com/jchamper/Fire-Ant-Suppression-Model).
