# Supplementary material for: Modeling Control of Invasive Fire Ants by Gene Drive
Source: Adv Sci (Weinh). 2025 Oct 27;12(46):e04653. doi: 10.1002/advs.202504653 (PMC12697894; doi:10.1002/advs.202504653)
Supplement: Supplementary file 1 — Supporting Information [file ADVS-12-e04653-s001.pdf]

Modeling control of invasive fire ants by gene drive

Yiran Liu[1], Samuel E. Champer[2], Benjamin C. Haller[2], Jackson Champer[1]*

[1]Center for Bioinformatics, Center for Life Sciences, School of Life Sciences, Peking University, Beijing, China

[2]Department of Computational Biology, Cornell University, Ithaca, United States

*jchamper@pku.edu.cn

Supplemental Information

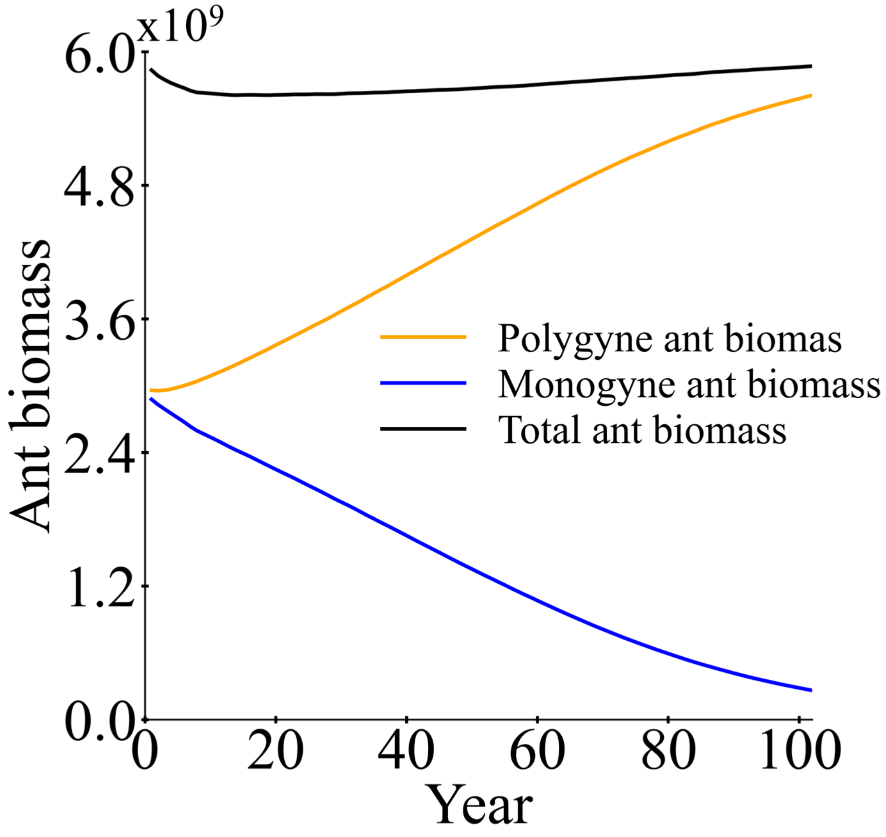

Figure S1 Total ant biomass without releasing drive. The total ant biomass in a mixed population (monogyne and polygyne) was recorded in each year without releasing drive individuals. One unit of biomass is the mass of one monogyne colony worker. Displayed data are the average of 20 simulations.

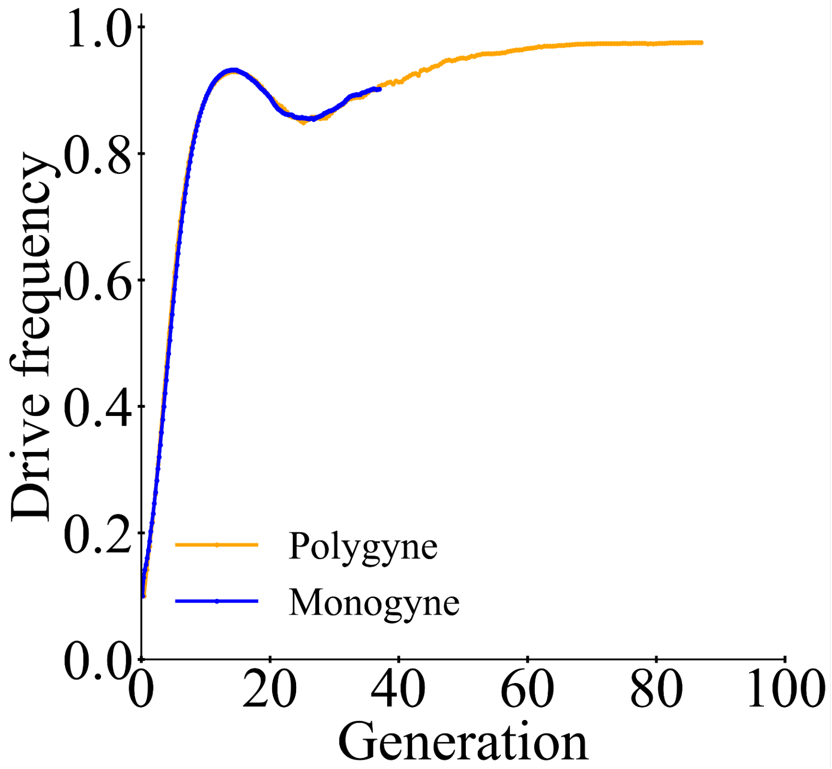

Figure S2 Comparison of generation time. The drive frequency in monogyne-only and polygyne-only models was tracked in each year. The initial population contained 10% drive heterozygous and 90% wild-type colonies. The simulation was run for a duration of 200 years, and these were converted to generations. For monogyne, the generation time was calculated as 5.41 years. The generation time for polygyne should be slightly higher than 2 years based on 50% queen replacement, and it was found that allele frequency would exactly match monogyne with a generation time of 2.3 years, as shown. Displayed data are the average of 200 simulations.

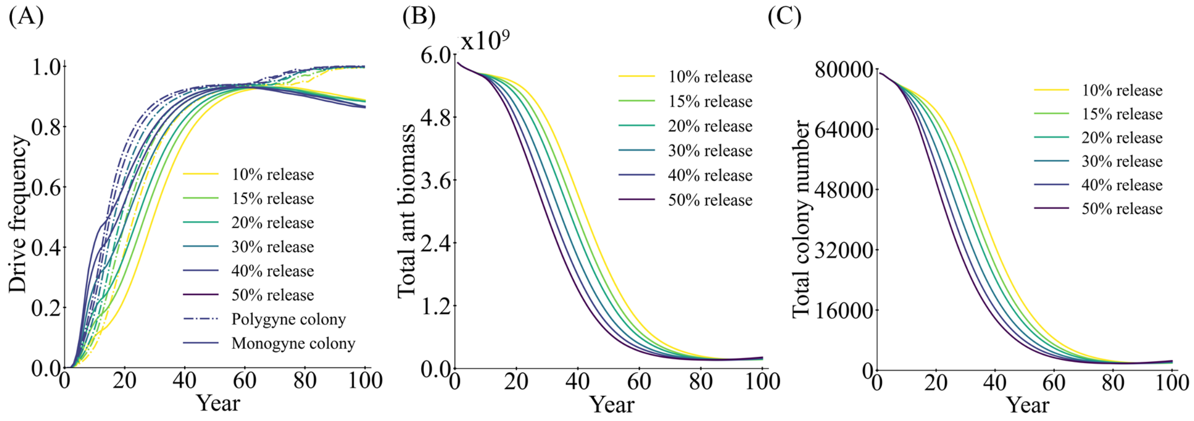

Figure S3 Effect of drive release size. With varying drive male release sizes over six years (as a fraction of the total population), we tracked the (**A**) drive frequency for polygyne (dashed lines) and monogyne (solid lines) social forms, (**B**) total ant biomass (in units equivalent to the size of an average monogyne colony worker), and (**C**) total colony number. Displayed data are the average of 200 simulations.

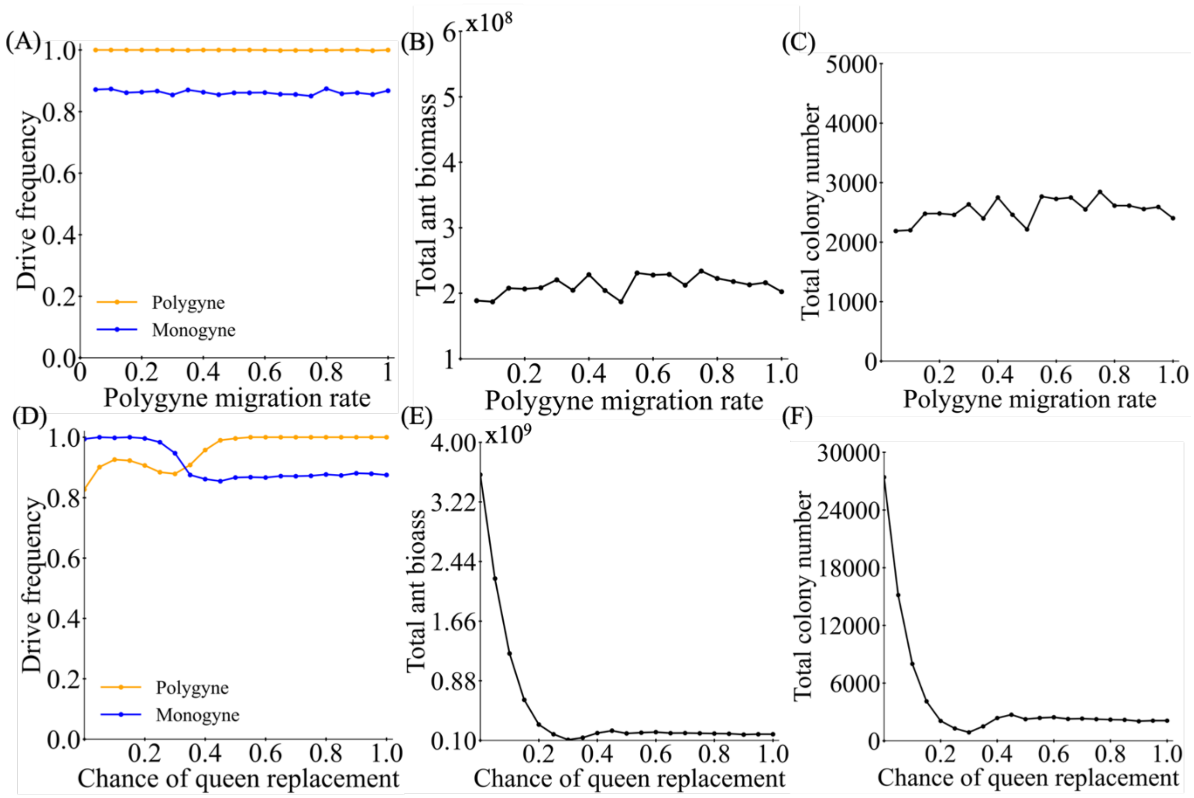

Figure S4 Suppression outcomes with varying polygyne colony parameters. We varied the (**A-C**) relative dispersal rate of polygyne ants compared to monogyne and varied the (**D-F**) chance of representative queen replacement in polygyne colonies. We tracked the (**A,D**) drive frequency, (**B,E**) total ant biomass (in units equivalent to the size of an average monogyne colony worker), and (**C,F**) total colony number. Displayed data are the average of 200 simulations.

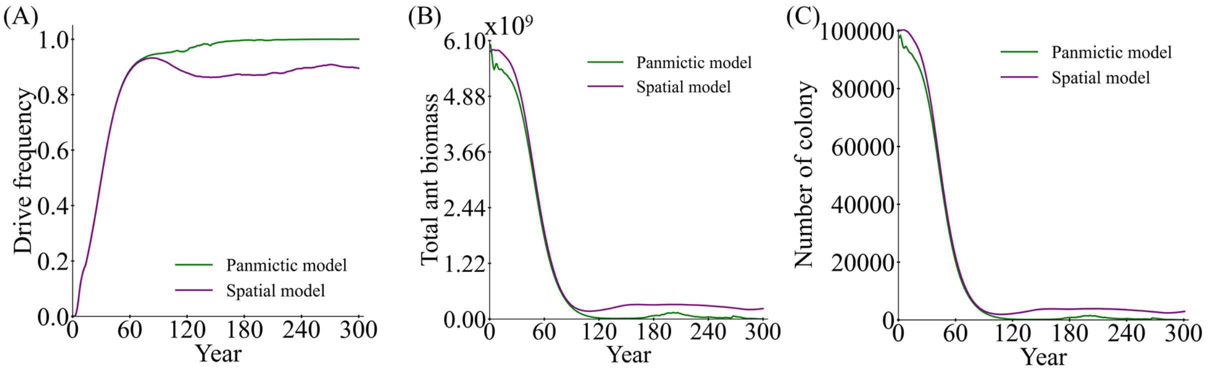

Figure S5 Comparison of panmictic and spatial fire ant models. Using default parameters and monogyne only populations in our normal spatial model and in a panmictic model, we tracked the (**A**) drive frequency, (**B**) total ant biomass (in units equivalent to the size of an average monogyne colony worker), and (**C**) number of colonies. Each point shows the average of 200 simulations.

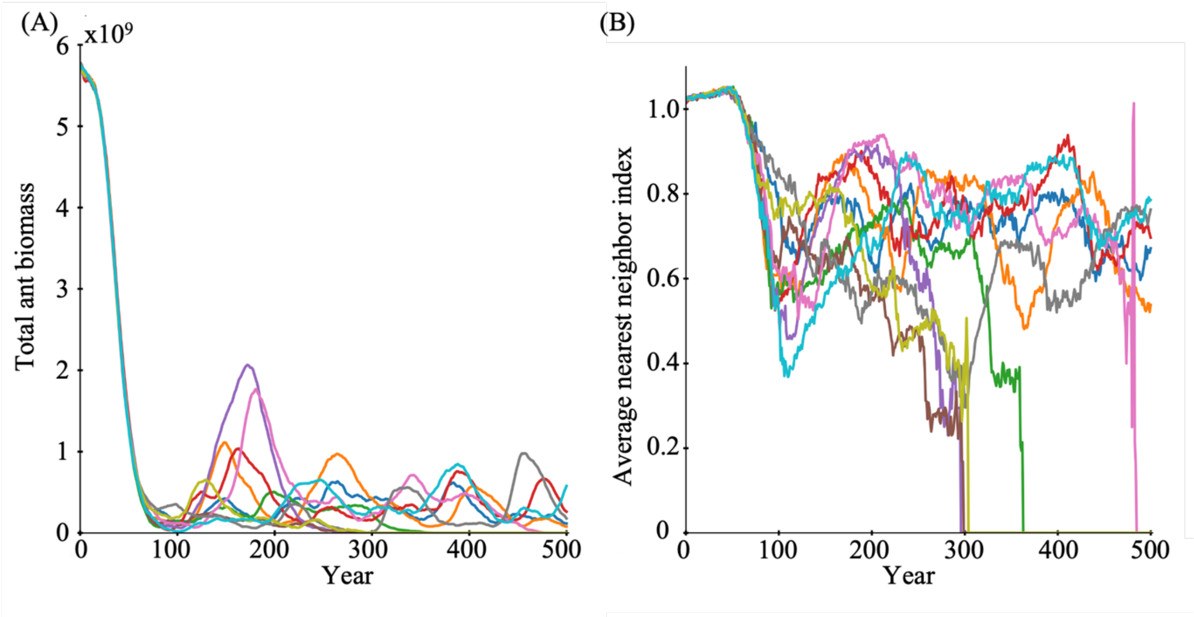

Figure S6 The chasing phenomenon in fire ant populations. Drive males were released into a spatial fire ant population. All model parameters were at their defaults, but the simulation was allowed to progress 500 years after the drive release. The average nearest neighbor ratio (low values < ~0.9 represent randomly distributed individuals, which is indicative of chasing if it remains low for several generations without population elimination) and total ant biomass (in units equivalent to the size of an average monogyne colony worker) were tracked in each year after releasing drive males. 10 independent simulations were conducted, with each line representing a distinct simulation trajectory.

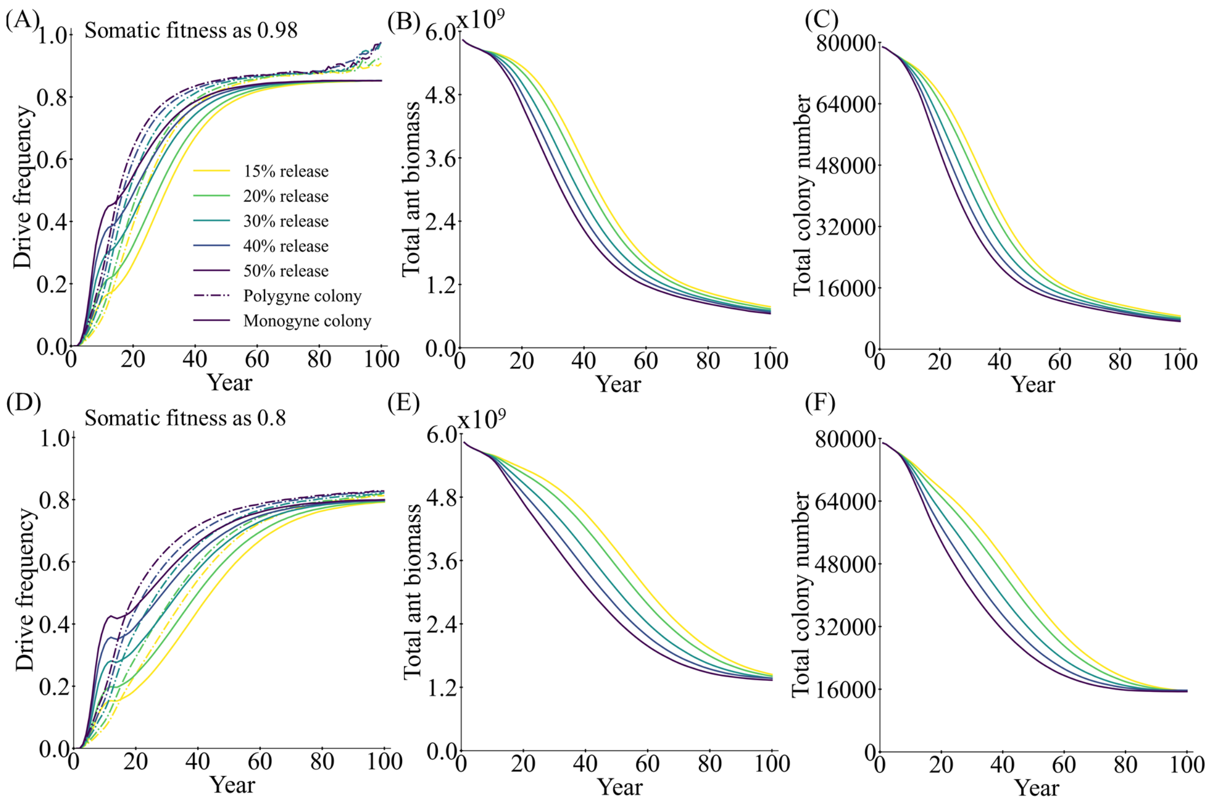

Figure S7 Different release ratios of dominant sterile resistance drive. In the fire ant model with a mixed spatial population (composed of a mix of monogyne and polygyne colonies), a drive conversion rate of 0.8, and a female fitness of (**A-C**) 0.98 or (**D-E**) 0.8, we release either standard or dominant-sterile resistance suppression drives. We track the (**A,D**) drive frequency, (**B,E**) ant biomass (in units equivalent to the size of an average monogyne colony worker), and (**C,F**) the number of colonies for monogyne and polygyne social forms. Displayed data are the average of 200 simulations.

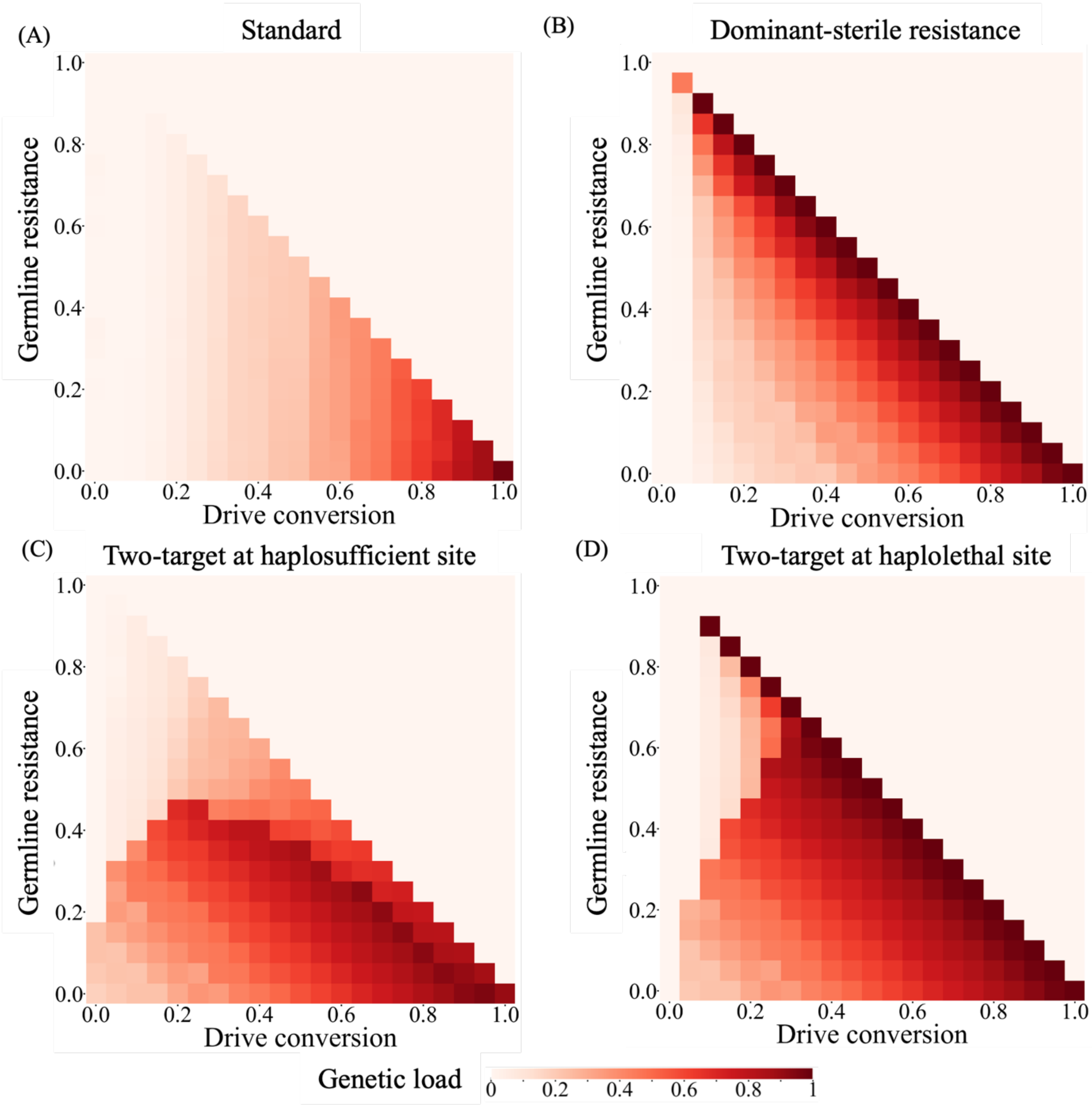

Figure S8 Suppression efficiency comparison of four suppression drive designs. Using a discrete-generation panmictic model, the equilibrium genetic load (suppressive power) is shown with default performance characteristics under varying drive conversion rate and germline resistance for (**A**) standard haplodiploid suppression drives targeting female fertility, (**B**) drives that produce dominant-sterile resistance alleles, (**C**) two-target design with the drive in a haplosufficient but essential gene, and (**D**) two-target design with the drive in a haplolethal gene. (A) and (B) are identical to Figure 5 to facilitate direct comparison. Data points in (C) and (D) are the average of 20 simulations.

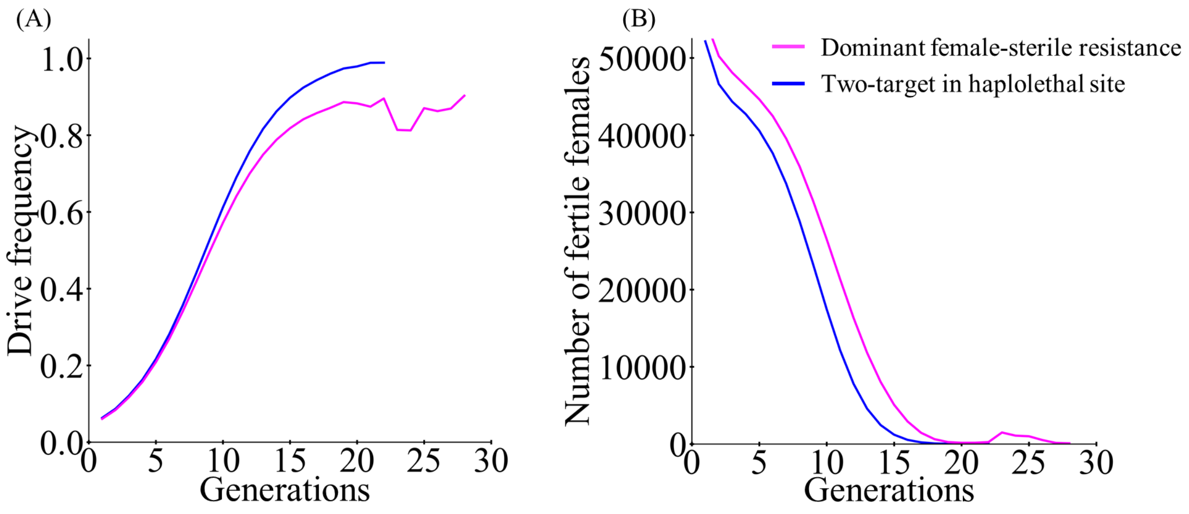

Figure S9 Comparison of haplolethal-suppression distant drive and dominant female-sterile resistance drive. Using the discrete-generation panmictic model, the **(A)** drive frequency and **(B)** population size in each generation are tracked with default performance characteristics, when total cut rate is 1. Displayed data are the average of 200 simulations.

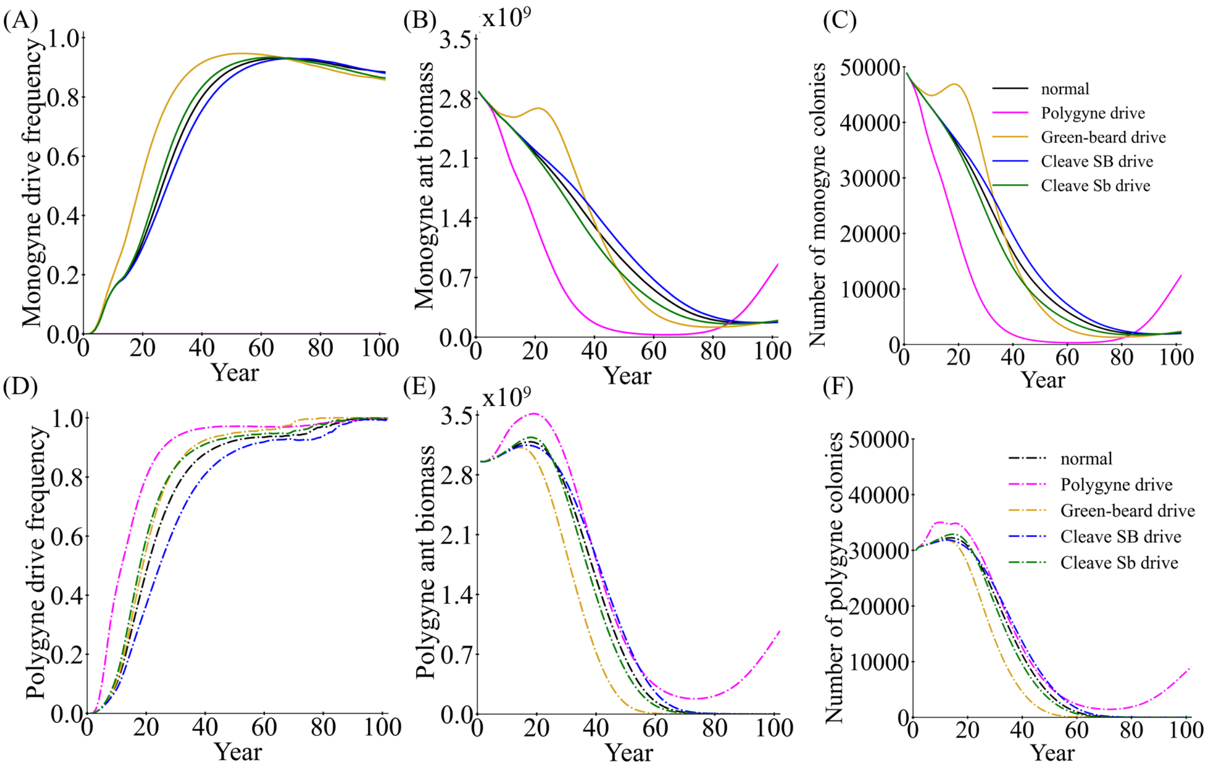

Figure S10 Comparison of new drive variants. Each drive variant was released into a mixed spatial fire ant population. For (**A-C**) monogyne and (**D-F**) polygyne social forms, we track the (**A,D**) drive frequency, (**B,E**) ant biomass (in units equivalent to the size of an average monogyne colony worker), and (**C,F**) number of colonies. Displayed data are the average of 200 simulations.

Octopamine β2 receptor

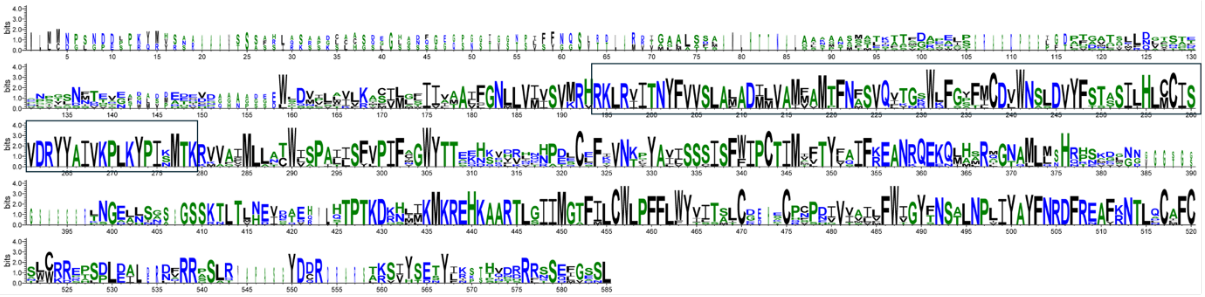

NADPH oxidase

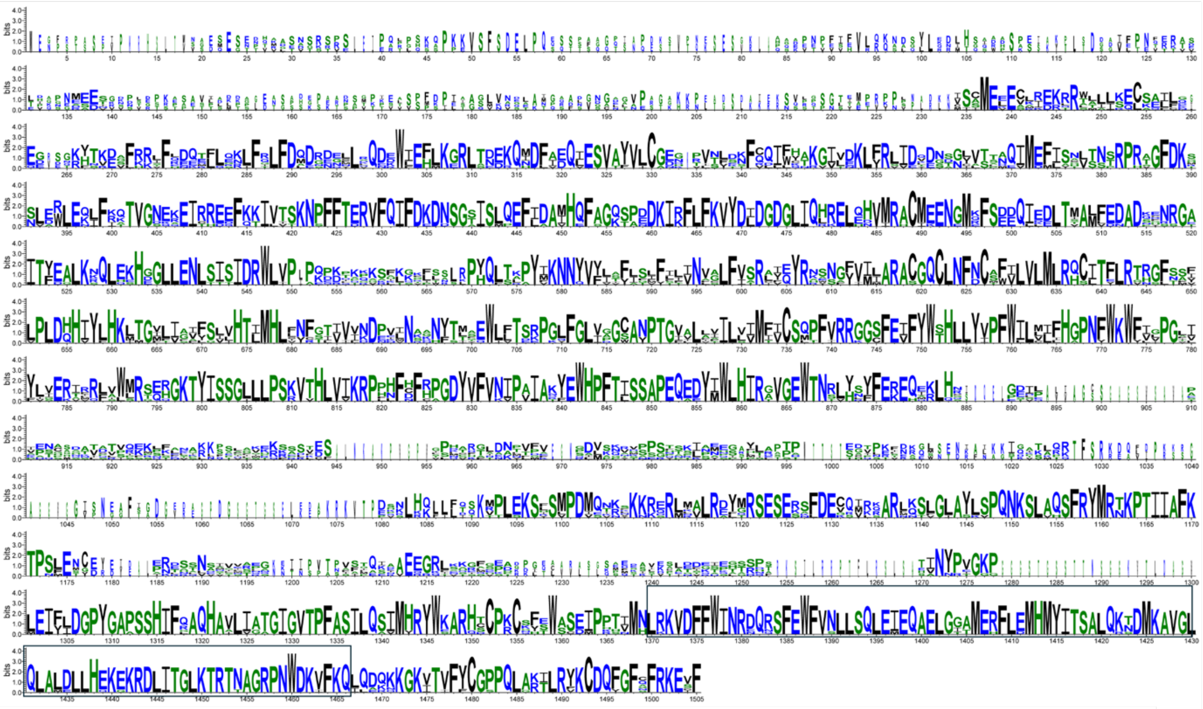

stall

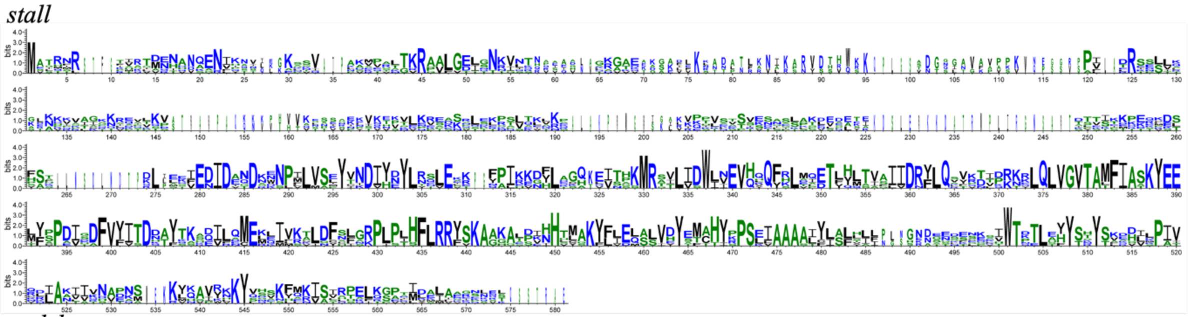

nudel

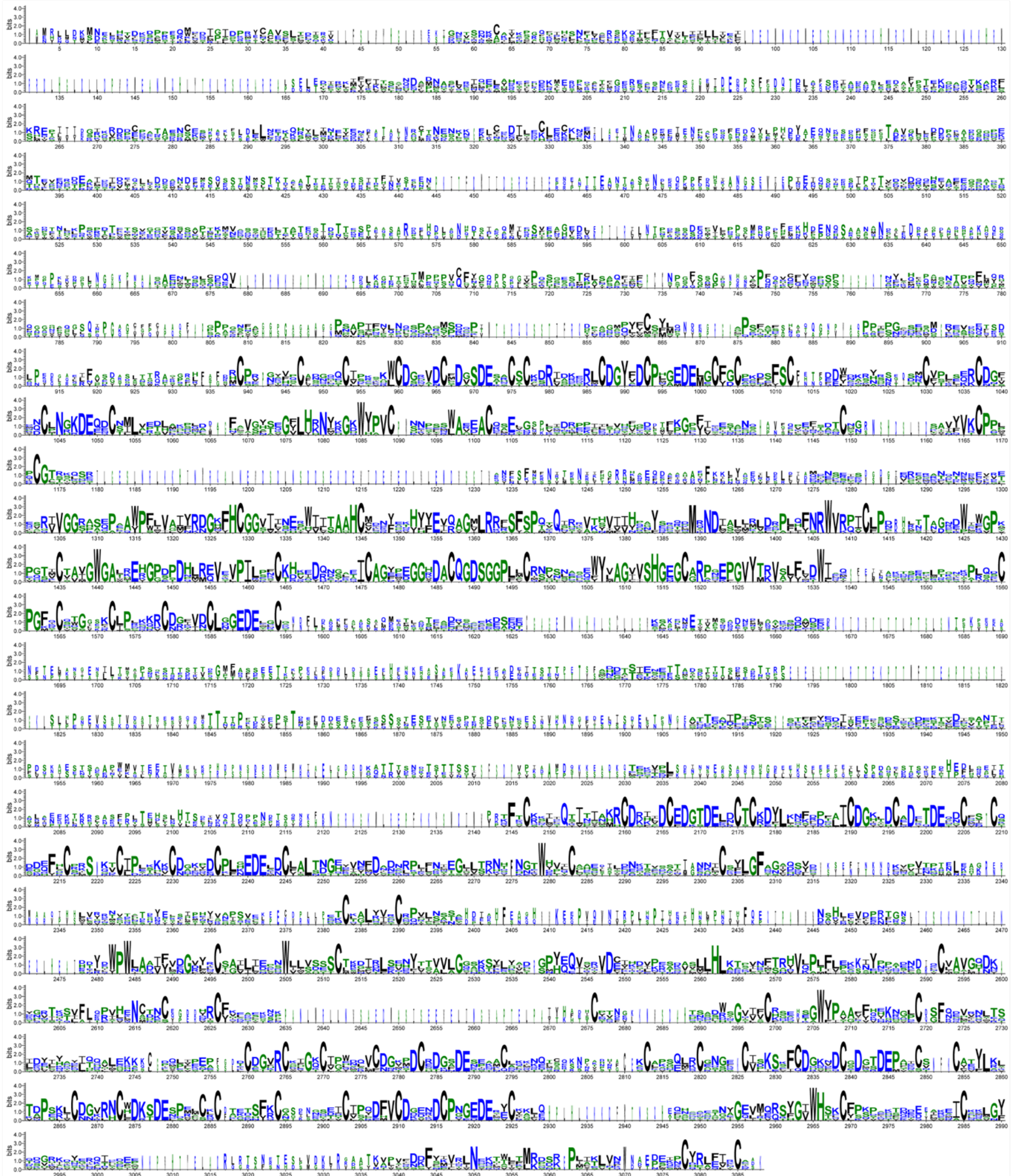

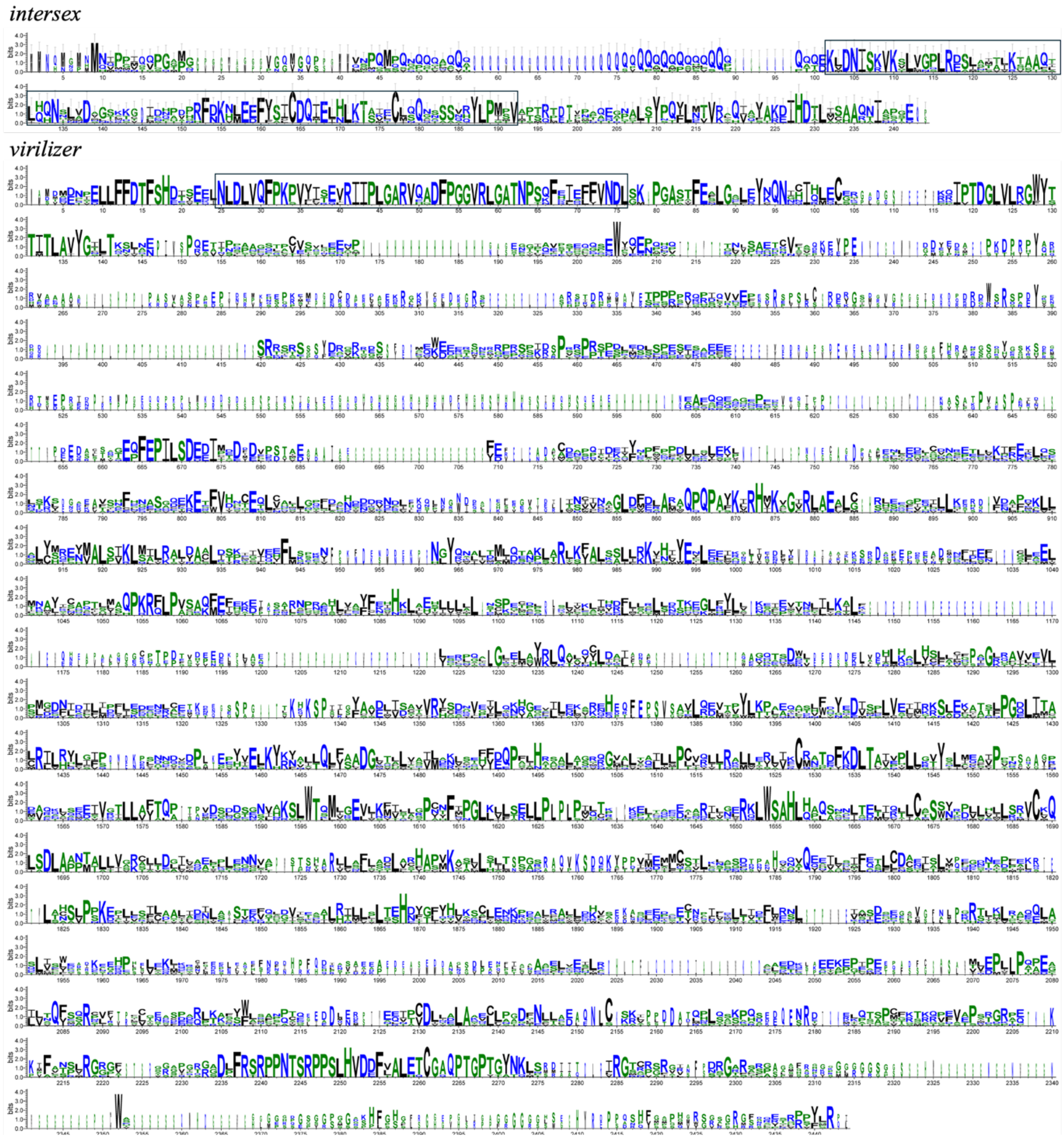

Figure S11 Multiple sequence alignment of candidate female fertility genes. Alignment includes sequences from *Solenopsis Invicta*, *Linepithema humile*, *Aedes aegypti*, *Anopheles stephensi*, *Drosophila melanogaster*, *Vespula vulgaris*, *Bemisia tabaci* and *Spodoptera frugiperda*. The region marked with a square is highly conserved at the animo acid level and was used for gRNA design (see Figure S11). Sequences for alignment were downloaded from the NCBI reference database. The online platform WebLogo (https://weblogo.berkeley.edu/logo.cgi) was used to visualize multiple protein sequence alignment. See next page for *stall* and *nudel* genes.

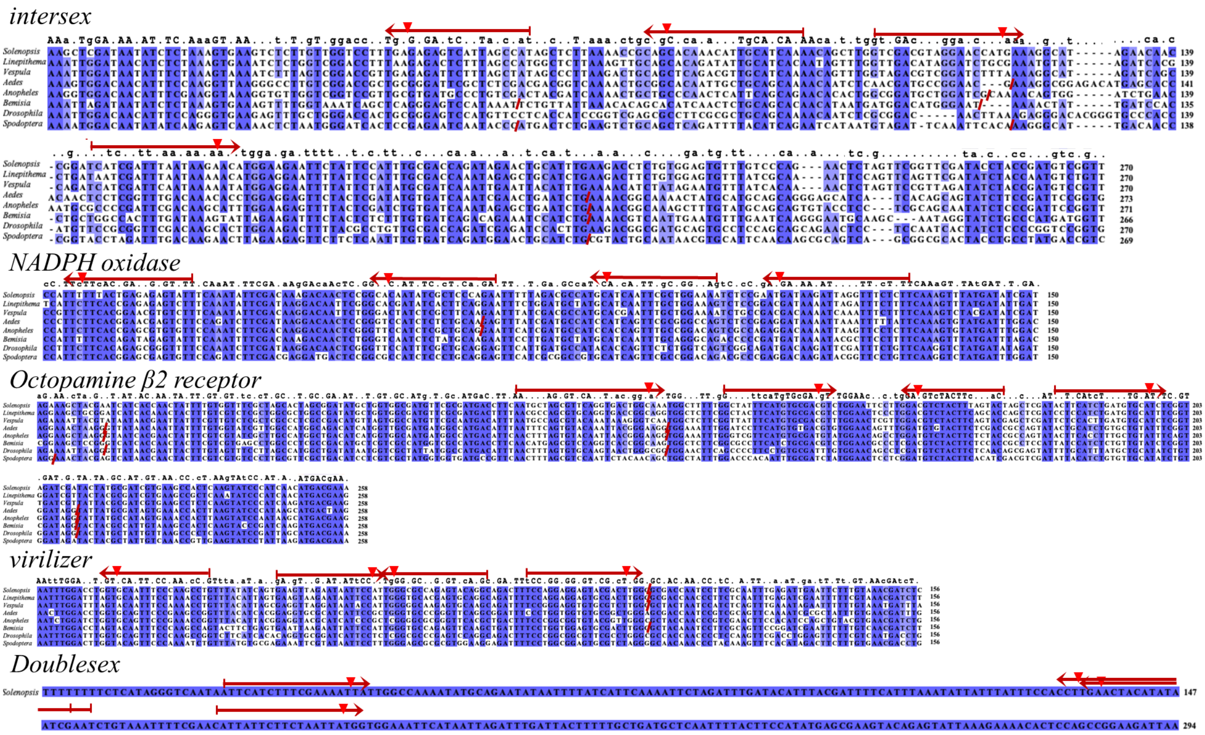

Figure S12 gRNA multiplexing targets in fire ant female fertility genes. Female fertility genes with highly conserved regions between several major insect species were identified. Within these regions, four fire ant target gRNA sequences suitable for a gRNA multiplexing strategy were identified using the Chopchop platform, indicated by red arrows. The gRNA cut sites were indicated by reversed triangle. The locations of introns in other species are labeled with red slashes.
